# Bimetallic lanthanide complexes that display a ratiometric response to oxygen concentrations†
†Electronic supplementary information (ESI) available: Synthetic procedures, luminescence and NMR spectra, photophysical considerations, time-resolved emission profiles and luminescence lifetimes. See DOI: 10.1039/c4sc03827d



**DOI:** 10.1039/c4sc03827d

**Published:** 2015-01-12

**Authors:** Thomas Just Sørensen, Alan M. Kenwright, Stephen Faulkner

**Affiliations:** a Chemistry Research Laboratory , University of Oxford , 12 Mansfield Road , Oxford OX1 3TA , UK . Email: stephen.faulkner@chem.ox.ac.uk; b Nano-Science Center & Department of Chemistry , University of Copenhagen , Universitetsparken 5 , DK-2100 København Ø , Denmark . Email: TJS@chem.ku.dk; c Department of Chemistry , University of Durham , South Road , Durham DH1 3LE , UK . Email: a.m.kenwright@dur.ac.uk

## Abstract

A pair of hetero-bimetallic lanthanide complexes containing terbium and europium ions have been prepared by coupling kinetically stable complexes together using an Ugi methodology to incorporate a naphthyl chromophore. Both complexes exhibit emission from terbium and europium in solution. The terbium centred emission varies with dissolved oxygen concentration, while the europium intensity remains essentially constant in one of the complexes.

## Abstract 2

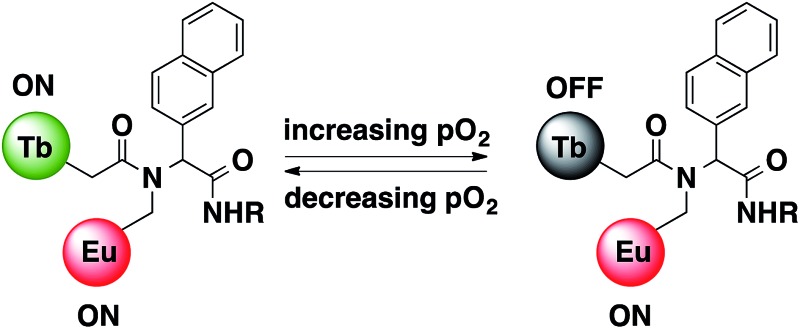
Heterometallic lanthanide complexes can display a ratiometric response to oxygen concentrations.

## Introduction

Oxygen has been known to be essential to life since the identification of the element, and oxygen levels play a key role in a wide range of biomedical problems. For instance, both heart disease and stroke are consequences of ischemia (oxygen starvation), while many solid tumours have hypoxic regions in which low oxygen levels change the cellular physiology. Despite this, hypoxia and ischemia are not fully understood, partly as a consequence of the difficulty in measuring oxygen concentrations in real time.^1–3^

Considerable effort has been devoted to the development of optical probes for oxygen concentration.^4–6^ These have tended to exploit the interaction between the triplet ground state of molecular oxygen and a triplet excited state.^7,8^ For instance the ^3^MLCT states of transition metal complexes, such as [Ru(Bpy)_3_]^2+^, are quenched by molecular oxygen, giving rise to a reduction in luminescence intensity with changes in dissolved oxygen concentration.^9–13^ Similarly, energy transfer from the triplet state of an organic chromophore to a lanthanide ion becomes reversible when the triplet state can be thermally repopulated from the lanthanide-centred excited state.^14–17^ When this occurs, the lanthanide emission will also vary with the concentration of dissolved oxygen. The chief issue with such monometallic probes is that only one chromophore is emissive. As such, it is impossible to tell whether weak emission arises from high oxygen concentration or low complex concentration. Parker and co-workers have used cocktails of europium and terbium complexes in which the terbium luminescence is quenched by oxygen but the luminescence from the europium ^5^D_0_ state is unaffected by oxygen concentrations.^6^

Our approach in this manuscript is different, and relies upon the use of molecular complexes with more than one emissive state.

In 2003, we showed that kinetically stable lanthanide complexes can be used as building blocks to access heterometallic lanthanide complexes.^18^ Since then we have extended this “kinetic control” approach to a wide range of polynuclear hetero-bimetallic and hetero-trimetallic architectures.^19–25^ In these systems, the emissive states of the different lanthanide ions are all populated from the same chromophore; the relative inefficiency of lanthanide to lanthanide energy transfer gives rise to multiple emissive states.

**Scheme 1:**
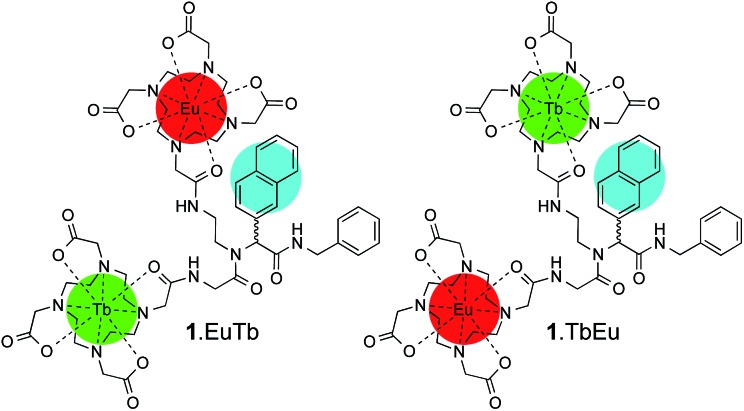
Structures of **1**.EuTb and **1**.TbEu.

Here, we study a pair of heterometallic complexes in which a naphthyl chromophore is used to sensitise terbium and europium, giving rise to oxygen-dependent luminescence from terbium and luminescence with reduced or negligible oxygen dependence from europium, depending on the relative rates of the processes involved in energy transfer from the chromophore. **1**.EuTb and **1**.TbEu (Scheme 1) show very different oxygen dependent photophysics as a direct consequence of the position of the metal centres in the molecular framework relative to that of a sensitizing chromophore.

## Results and discussion

### Rationale

**Fig. 1:**
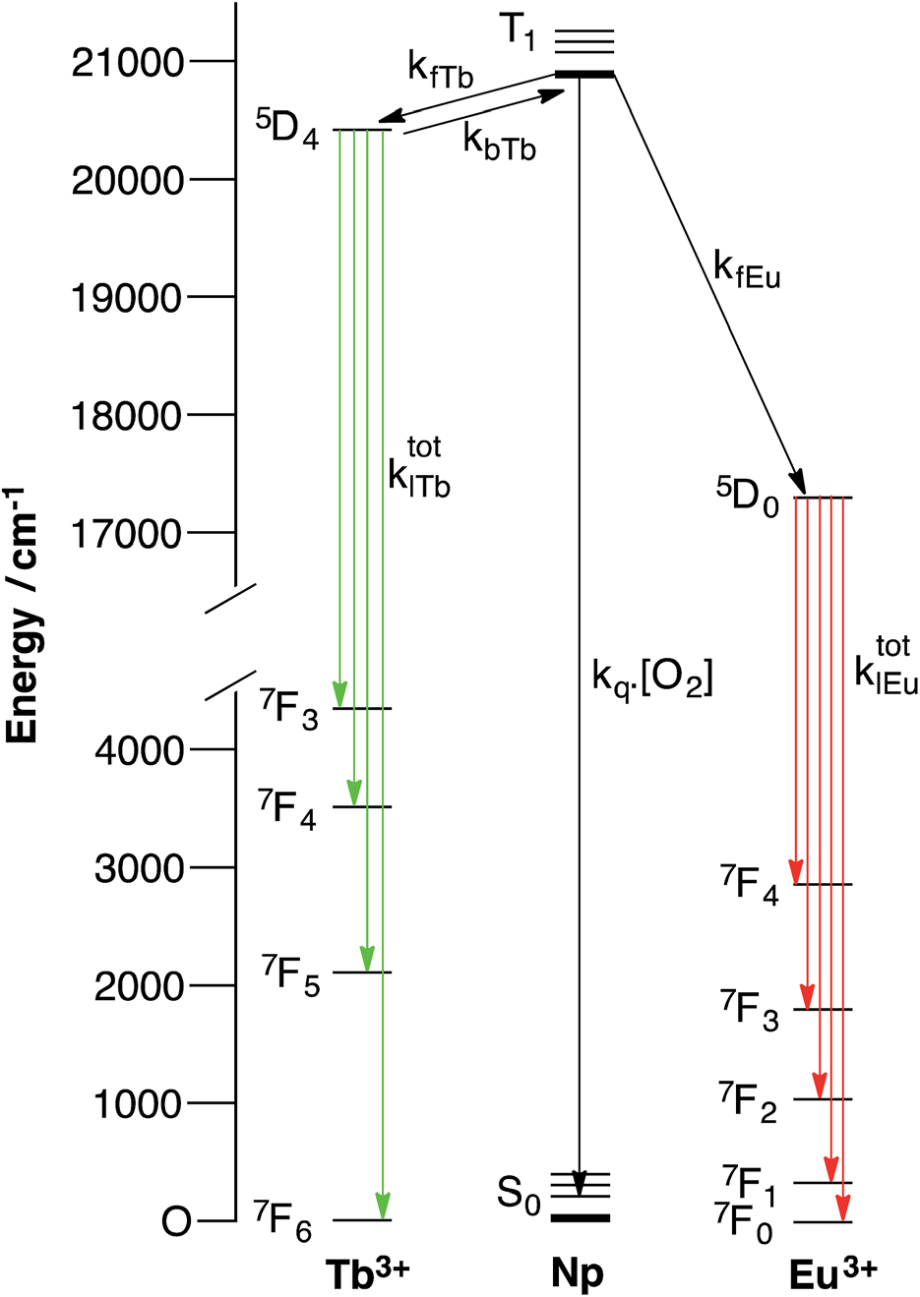
Simplified energy level diagram showing the key processes in a bimetallic system containing a europium ion, a terbium ion and a naphthyl chromophore (Np). For both lanthanide ions, a partial ground state manifold is shown in the interests of simplicity (NB back energy transfer from europium can be neglected).

If we consider a complex of the form ArLEuTb, a number of possible pathways can give rise to metal-centred luminescence. The emissive states of terbium (^5^D_4_, 20 490 cm^–1^) and europium (^5^D_0_, 17 240 cm^–1^) are both populated through the triplet states of aryl chromophores (Ar). However, when naphthyl chromophores (Np) are used as sensitizing antennae, the triplet state of the naphthyl group (20 850 cm^–1^) can be thermally repopulated from the terbium ^5^D_4_ state. In a heterometallic system, a complex range of processes can therefore govern the emission from both europium and terbium centres. These are summarized in Fig. 1, which shows how collisional quenching with oxygen can deactivate the triplet state (see ESI† for details). In addition, the rate of energy transfer from the chromophore to the lanthanide can have a profound effect on the oxygen dependence of lanthanide luminescence. We recently showed how important such effects could be by varying the separation between the donor chromophore and the lanthanide acceptor in mononuclear systems which combined pyrene antennae and europium centres.^26^ In these, it was found that reversible energy transfer and pre-equilibration were important at short donor–lanthanide separations (where both forward and back energy transfer are relatively rapid), while systems with larger donor–lanthanide separations exhibit oxygen dependence as a consequence of competitive quenching of the aryl triplet state and relatively slow forward energy transfer. In the latter case, pre-equilibration does not occur. As such, there will always be occasions where a chromophore can sensitise luminescence from a lanthanide reversibly, though this need not invariably be the case. Therefore, pre-equilibration and competitive quenching of the triplet state both offer potentially valid mechanisms for achieving oxygen responsive lanthanide emission, while variation in separation can be exploited to mediate the degree of response to oxygen.

**Scheme 2:**
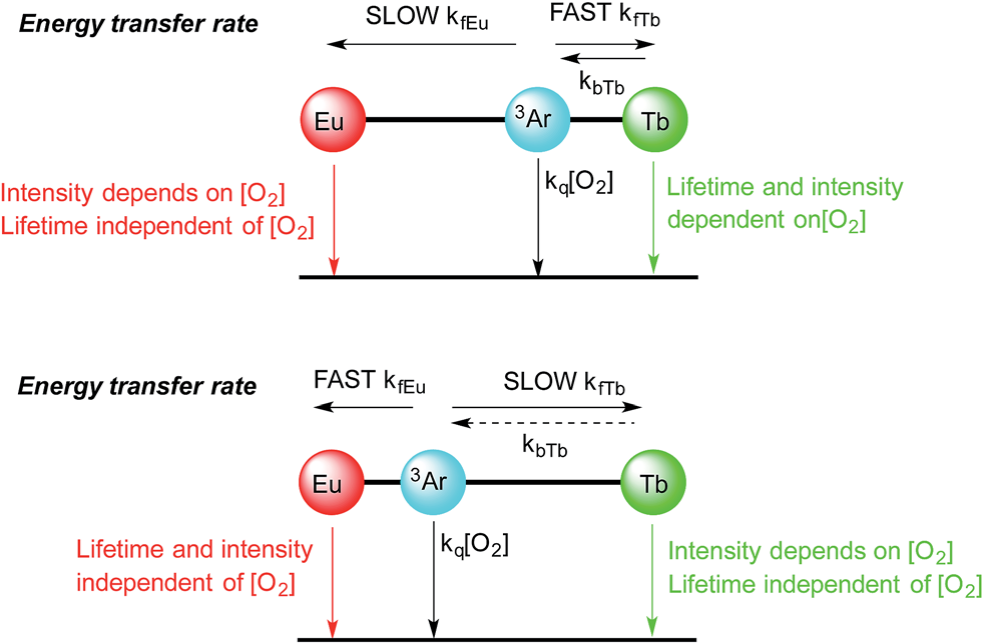
Cartoons showing the effect of aryl chromophore lanthanide separation on the dependence of oxygen emission, (top) with short Ar–Tb and long Ar–Eu separation, (bottom) with long Ar–Tb and short Ar–Eu separation.

For instance in Scheme 2 (bottom), the cartoon shows a large separation between a terbium centre and the sensitizing chromophore (Ar), which in turn is relatively close to the europium centre. In this case, energy transfer to terbium will be slow, resulting in oxygen dependence of the terbium centred luminescence without a significant change in terbium luminescence lifetime, while energy transfer to europium occurs relatively quickly (as consequence of the smaller Ar–Eu separation) and gives rise to a much lower dependence of the europium centred luminescence on oxygen.

In Scheme 2 (top), the positions of the lanthanides are reversed so that the small Ar–Tb separation, and consequently fast energy transfer, will increase the rate of energy transfer to terbium. In this case, the increased Ar–Eu separation will give rise to oxygen dependence of the europium centred signal as a consequence of relatively slow energy transfer. It should be noted that the lifetime of the europium centre would be expected to be independent of oxygen concentration in both cases, since back energy transfer by thermal repopulation is precluded by the large separation between the chromophore triplet energy and the ^5^D_0_ emissive state of europium(iii).

### Synthesis

**Scheme 3:**
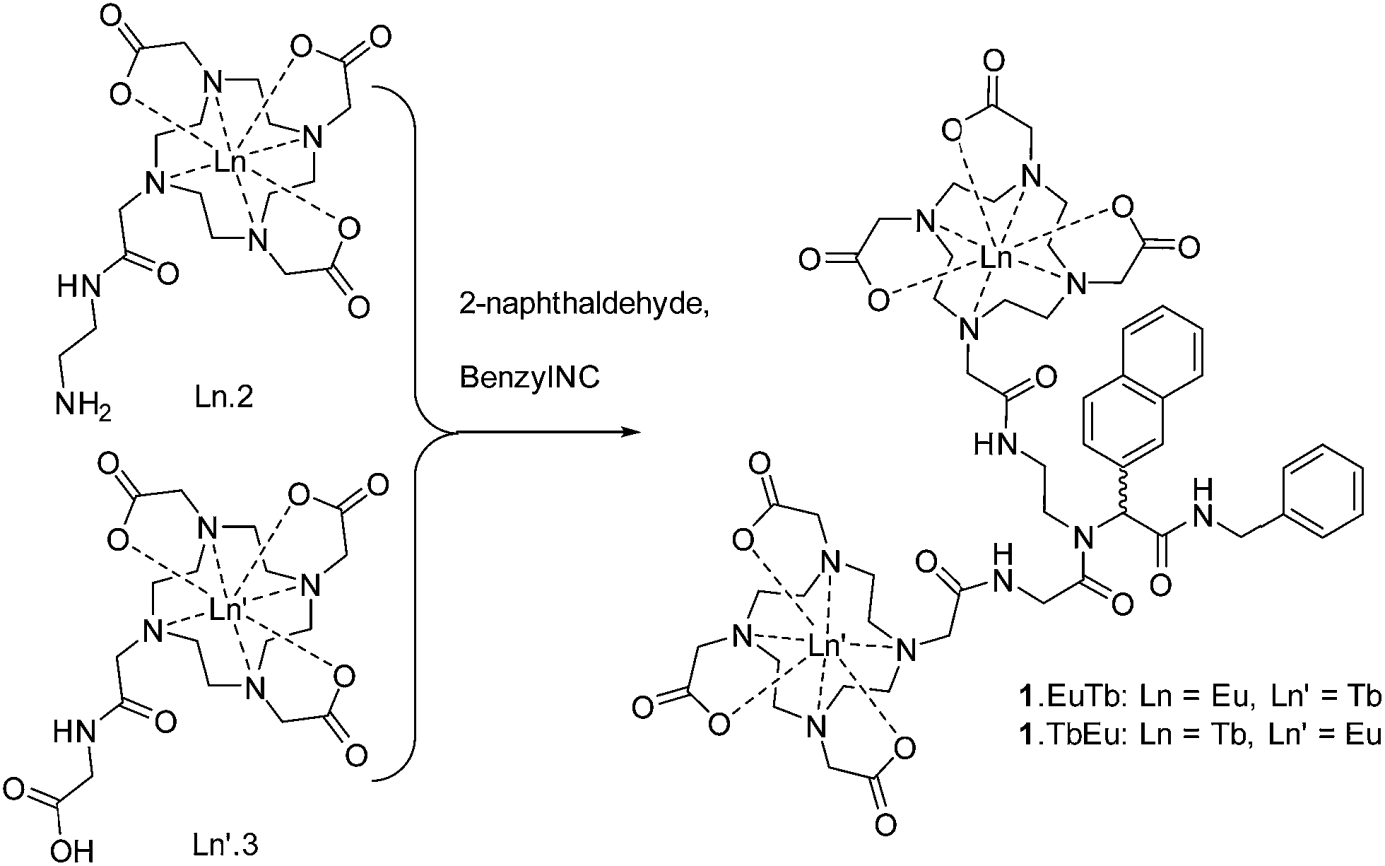
Synthesis of **1**.LnLn′ complexes.

With this complexity in mind, we resolved to investigate the luminescence properties of two lanthanide complexes (**1**.EuTb and **1**.TbEu) in which the same ligand backbone is used to explore the role of the position of both metal ions. These were prepared by reaction of Ln.2, Ln′3 and 2-naphthaldehyde with benzylisonitrile as shown in Scheme 3 (further details can be found in the ESI† to this paper).

**Fig. 2:**
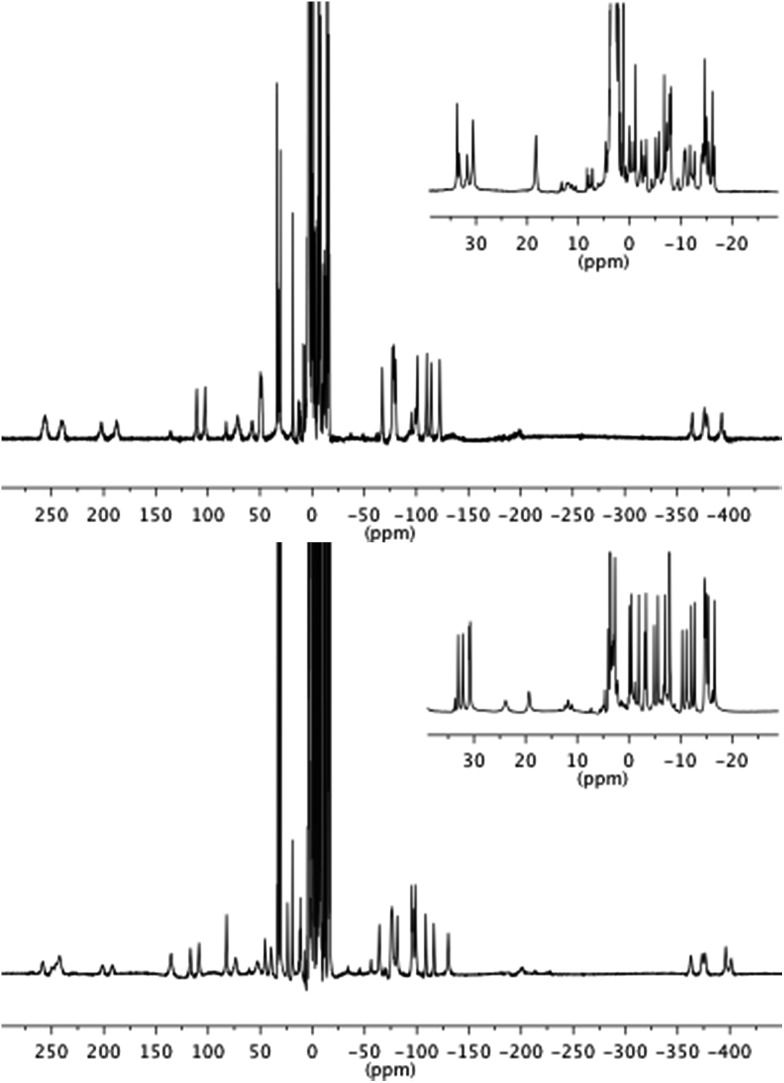
^1^H NMR spectra of **1**.EuTb (top) and **1**.TbEu (bottom).

Both **1**.EuTb and **1**.TbEu gave ^1^H NMR spectra consistent with their structures. Furthermore it is clear that the metals are in different binding pockets from the spectra (Fig. 2): for instance, consideration of the resonances corresponding to the cyclen ring axial protons for the terbium binding site (in the range –380 to –400 ppm) shows that the terbium coordination environments are different (though broadly similar) in **1**.EuTb and **1**.TbEu.

Although the NMR spectra of the bimetallic complexes are wholly consistent with the reported structures and show broad agreement in the relevant regions with the spectra of the monometallic precursors, it is not possible to make a detailed comparison of the peaks in the two sets of spectra (monometallic and bimetallic) because, for complexes that are not axially symmetric, the peak positions in the spectra depend on both the axial and rhombic terms of the magnetic anisotropy,^27^ and those terms will change unpredictably on coupling of the monometallic complexes. Thus, while it might be tempting to interpret peaks that are not greatly shifted (say in the region +35 to –35 ppm) solely in terms of them arising from hydrogens close to the Eu end of the complex, it is unsafe to do so without corroborating evidence (*e.g.* COSY), which is not readily available given the linewidths observed. Further, it should be noted that the number of potential isomers observable doubles on coupling of the two monometallic complexes as a consequence of the potential for diastereoisomerism.

### Photophysical properties

**Fig. 3:**
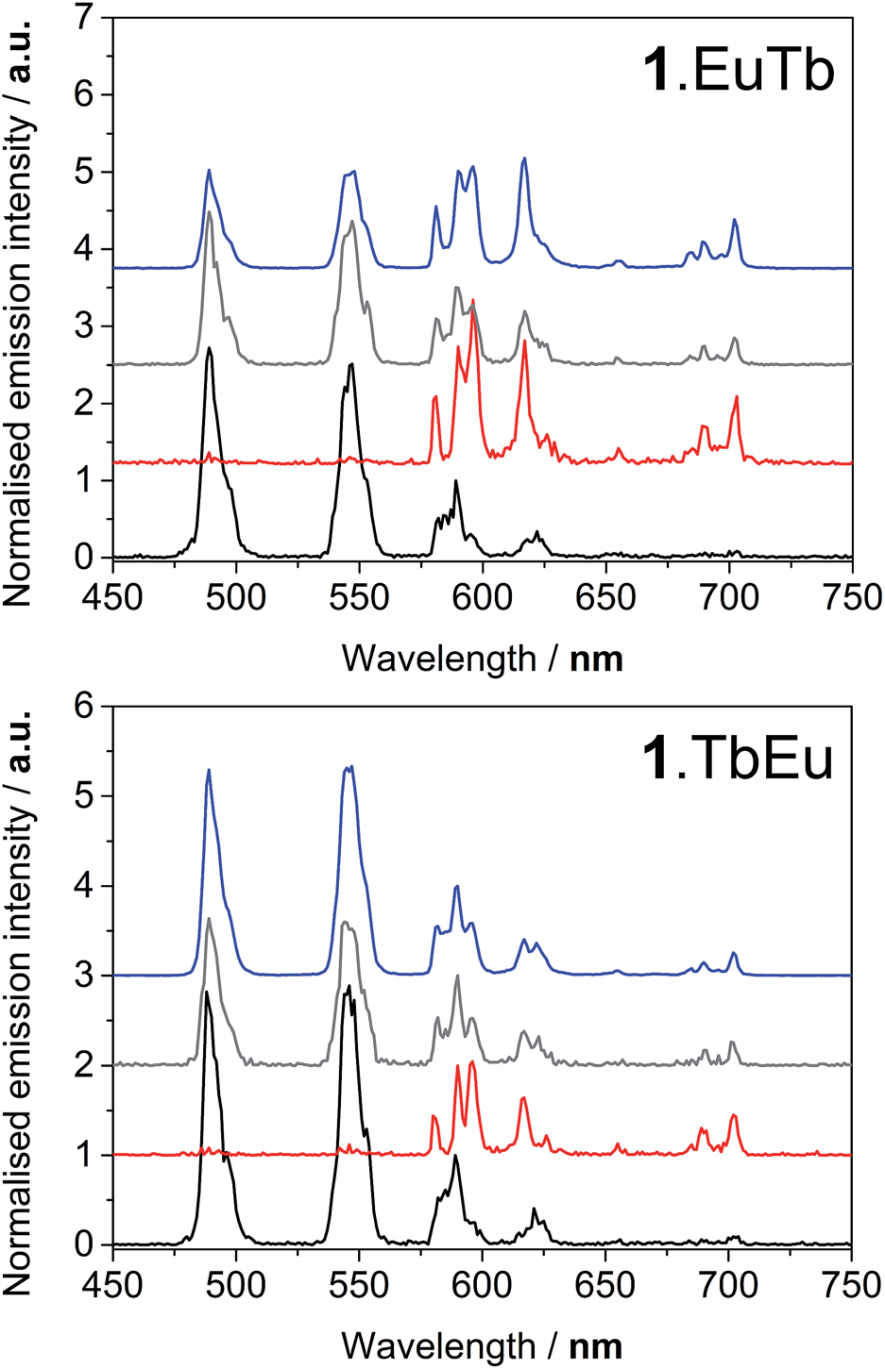
Emission spectra of **1**.EuTb (top) and **1**.TbEu (bottom) following excitation at 290 nm (blue), 380 nm (grey), 392 nm (red) and 488 nm (black).

Upon excitation of the naphthyl chromophore at 290 nm, both lanthanide ions exhibited sensitised emission. Excitation spectra obtained by observing the emission intensity at 545 nm and 700 nm (corresponding to the Tb(iii) ^5^D_4_–^7^F_5_ and Eu ^5^D_0_–^7^F_4_ transitions respectively) revealed that the naphthyl chromophore was clearly implicated as an antenna group for both lanthanides (see ESI†). Under ambient oxygen conditions, excitation of the naphthyl absorption band at 290 nm, gave rise to emission from both lanthanides in both **1**.EuTb and **1**.TbEu (Fig. 3). However, the relative intensity of terbium and europium luminescence varied markedly between the two complexes, with **1**.EuTb exhibiting similar peak intensities for the most intense transitions for the Eu^3+^ and Tb^3+^ ions. By contrast, in **1**.TbEu, the emission observed from Tb^3+^ was markedly more intense. At the concentrations used in this study, no emission from naphthyl excimers was observed; it is assumed that the steric bulk of the molecules prevent the close contact needed to form the excited state complexes.^28,29^

Excitation of the terbium centre through the f–f excited state manifold in both complexes resulted entirely in terbium-centred emission, while direct excitation of the europium centre resulted solely in europium centred emission (Fig. 3). Furthermore, the intensity ratio of the hypersensitive Δ*J* = 2 transition at 619 nm to that of the Δ*J* = 1 transition around 595 nm varied markedly between **1**.EuTb and **1**.TbEu, confirming the localisation of europium in different binding pockets within the ligand. Excitation at 380 nm results in population of excited states in both terbium and europium, and gave rise to very similar Tb : Eu intensity ratios for both complexes. This observation is remarkably different to that obtained by indirect excitation through the chromophore, and constitutes the first clear evidence that energy transfer can determine the form of the spectra in these bimetallic arrays (as discussed above and in Scheme 2). It is likely that sensitization occurs through space, since there is no suitable through-bond pathway for Dexter exchange. The observations from the steady state spectra were suggestive that, under ambient conditions, the rate of energy transfer for a given lanthanide was highly dependent on the position in which the lanthanide was bound. This lends credence to the hypothesis presented in the cartoon representation in Scheme 2, and implies that energy transfer to the two different binding sites occurs at very different rates as a consequence of differing lanthanide–chromophore separations. Thus it is clear that the two lanthanide centres must be situated very differently relative to the chromophore.

In fact, and unlike the cartoons in Scheme 2, **1**.LnLn′ can exist in a range of conformations—the flexibility of the backbone and the possibilities for bond rotation mean that both centres will exist in a wide range of conformational space; it is certain that different conformers will give rise to different relative intensities of emission from the terbium and europium centres (see ESI†). As a result, the terbium and europium emission spectra will reflect different weighted averages of the available conformations.

From the excitation spectra, the ratio of the intensity observed when exciting the terbium centre directly to that observed when exciting the naphthyl chromophore (*I*_Lm,Tb_(Np)/*I*_Lm,Tb_(direct)) can be determined. In cases where energy transfer is relatively slow, the overall intensity of the total emission spectrum will reflect the rate of energy transfer if competing processes can quench the intermediate triplet state. Thus the ratio of intensities will reflect the rate of energy transfer, with faster energy transfer processes being reflected in larger ratios, while slower energy transfer processes are reflected in smaller ratios. For **1**.EuTb the ratio (*I*_Lm,Tb_(Np)/*I*_Lm,Tb_(direct)) is 2.2, while for **1**.TbEu it is 5.6. Thus naphthyl is significantly more effective in sensitising terbium in **1**.TbEu than in **1**.EuTb relative to direct excitation. The same exercise can be done for europium. In this case the ratio (*I*_Lm,Eu_(Np)/*I*_Lm,Eu_(direct)) is 1.4 for **1**.EuTb and 0.5 for **1**.TbEu.

This implies that energy transfer from naphthyl to terbium occurs more rapidly than to europium in **1**.TbEu. In **1**.EuTb, the case is altered, and transfer to europium is much more competitive. These results would imply that the dominant conformer in both cases is the one in which the Ln centre in **1**.LnLn′ is closer than the Ln′ centre to the donor chromophore (see Schemes 1 and 3). The relative ratios will, of course, also reflect the better spectral overlap between the naphthyl triplet state and the terbium absorption spectrum, leading to more effective sensitization of terbium than europium in both cases.

**Fig. 4:**
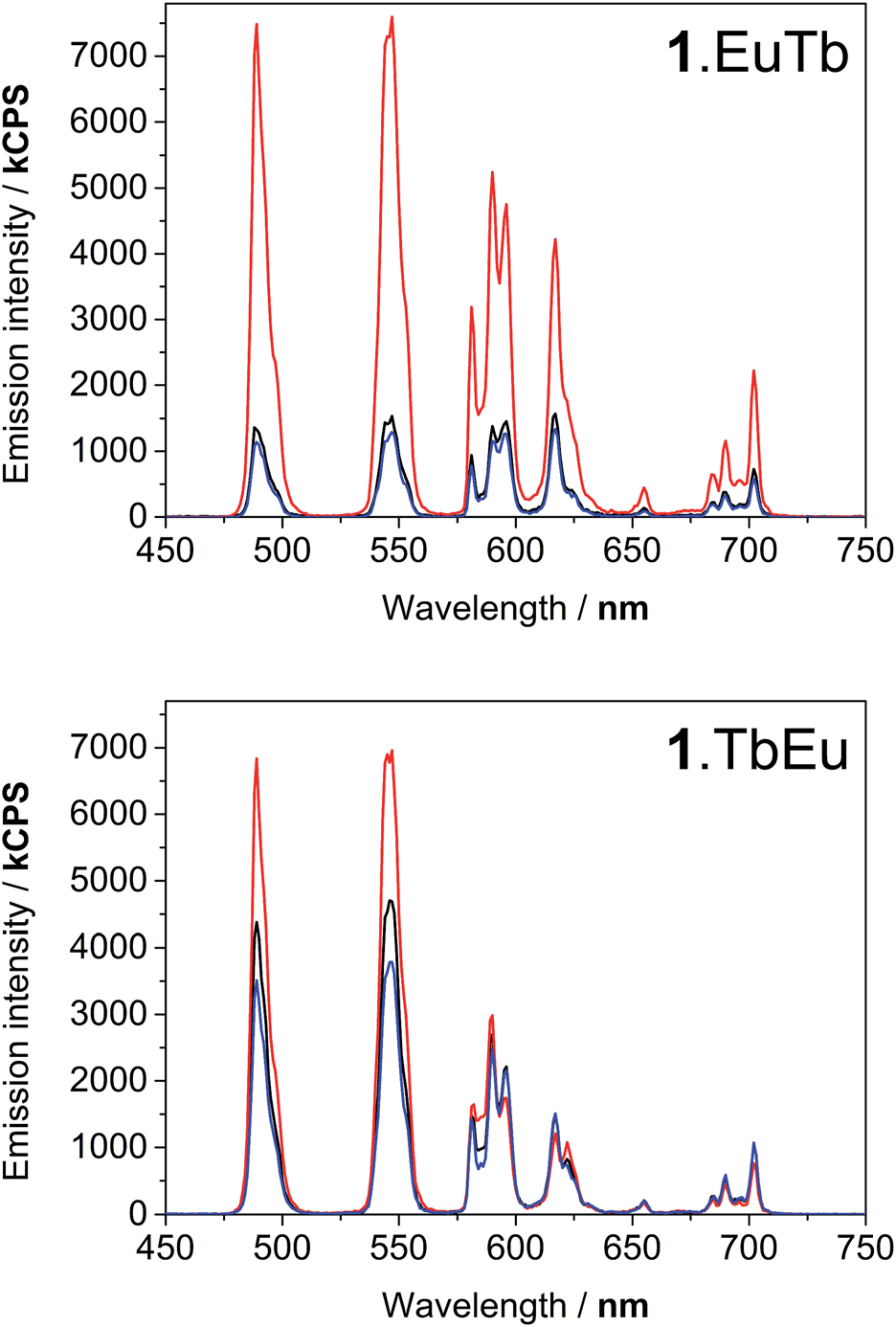
Luminescence spectra of **1**.EuTb (top) and **1**.TbEu (bottom) following excitation at 290 nm, showing the response to oxygen concentration: degassed (red), ambient (black), and oxygen saturated (blue). All spectra are uncorrected.

Degassing the samples resulted in a dramatic enhancement of the terbium-centred luminescence relative to that of the europium ion in both cases. Fig. 4 illustrates the nature of this change, and shows that there are clear differences between the behaviour of **1**.EuTb and **1**.TbEu. In the case of **1**.TbEu, there are dramatic variations in the intensity of the terbium luminescence but europium centred luminescence is essentially unchanged with changes in the oxygen concentration. By contrast, in **1**.EuTb, the intensity of the luminescence from both ions varies, albeit to differing degrees, as a function of oxygen concentration. Such behaviour can be explained by further consideration of the structure of the complexes, and is consistent with much slower energy transfer to Eu^3+^ in **1**.TbEu than in **1**.EuTb, implying greater chromophore to lanthanide separation in the former case.

Time-resolved luminescence lifetime measurements under ambient and degassed conditions provided further insight into the processes involved in energy transfer. As the data in Table 1 and S1–5† show, it is immediately apparent that the europium and terbium centres in both complexes exist in very similar environments (as might be expected when all are derived from DOTAmonoamide derivatives). More importantly, the measured luminescence lifetimes in aerated and degassed aqueous solution were the same within error for all the lanthanides studied. In the case of europium this is not unexpected, since thermal repopulation of the naphthyl T_1_ state is not feasible. However, in the case of the terbium complexes these observations clearly imply that back energy transfer from terbium to the naphthyl triplet state does not play a significant role in giving rise to oxygen dependence, since any pre-equilibration between the terbium excited state and the triplet state would be expected to reduce the observed luminescence lifetime of the terbium emission. As such, it is clear that the differences in the steady state spectra described above can all be ascribed to variations in the rate of energy transfer between the different centres.

**Table 1:** Luminescence lifetimes of **1**.EuTb and **1**.TbEu

		*τ*_H_2_O_ (Tb)^*a*^/ms	*τ*_D_2_O_ (Tb)^*b*^/ms	*q* (Tb)^*c*^	*τ*_H_2_O_ (Eu)^*b*^/ms	*τ*_D_2_O_ (Eu)^*b*^/ms	*q* (Eu)^*c*^
**1**.EuTb	Aerated	1.88	3.15	0.8	0.60	2.10	1.1
	Degassed	1.88			0.62		
**1**.TbEu	Aerated	1.78	3.10	1.1	0.64	2.43	1.1
	Degassed	1.80			0.63		

^*a*^Calculated by measuring the luminescence lifetime at 545 nm.

^*b*^Calculated by measuring the luminescence lifetime at 690 nm.

^*c*^Calculated using the method described in ref. 30; all luminescence lifetimes are ±10%.

These time-resolved observations bear out our early conclusion about relatively slow energy transfer in the case of **1**.EuTb, confirming the supposition of greater Tb–Np separation in this complex. In the case of **1**.TbEu, they provide additional information; clearly showing that, even though the terbium centre is closer to the naphthyl group, energy transfer between the two is still not sufficiently quick to allow any significant degree of equilibration between the two states. Thus it is clear that, although closer in space, the lanthanide chromophore separation is still greater than that required to achieve pre-equilibration.

### The effect of varying oxygen concentration

**Fig. 5:**
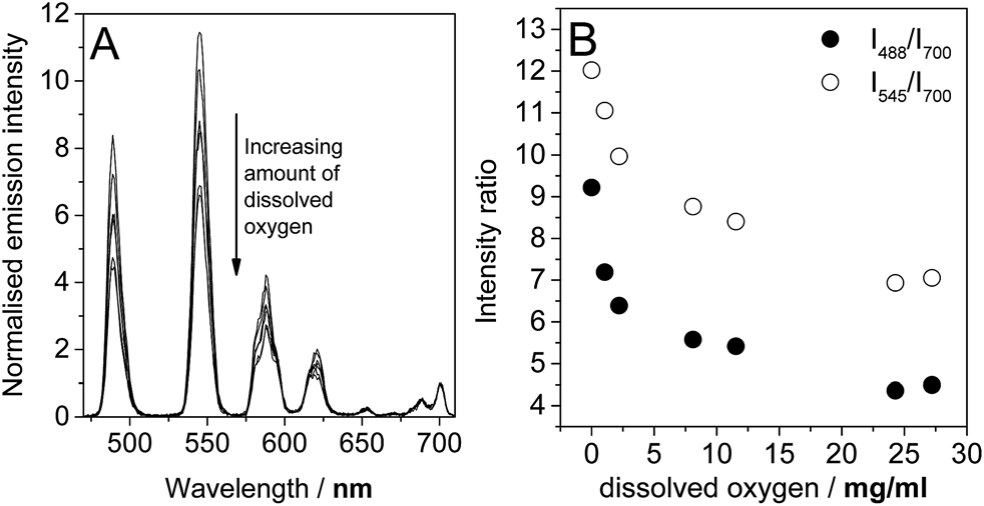
Variation in (A) the time-gated luminescence spectra upon excitation at 290 nm and (B) the ratios of the intensities of the terbium centred emission (at 488 or 545 nm) to that of the europium centred emission (at 700 nm) with oxygen concentration in water.

In the light of the photophysical measurements described above, it was resolved to study the variation in lanthanide luminescence with oxygen concentration. Using an oxygen electrode (Mettler Toledo InLab® OptiOx) to quantify oxygen concentrations in solution, we carried out a series of measurement of the luminescence spectra of **1**.TbEu at a range of dissolved oxygen concentrations. **1**.TbEu was chosen as the candidate for this study because the europium luminescence had already been shown to be invariant between degassed and aerated solution, meaning that the europium luminescence could potentially provide a reference signal that allows quantification of oxygen with a luminescent probe. The spectra obtained are shown in Fig. 5. As anticipated the luminescence from terbium following excitation at 290 nm varies dramatically with the concentration of dissolved oxygen while that of europium remains essentially constant. Fig. 5b shows the ratios of the intensities of the terbium ^5^D_4_–^7^F_6_ and ^5^D_4_–^7^F_5_ transitions (at 488 nm and 545 nm respectively) to that of the europium ^5^D_0_–^7^F_4_ transition (at 700 nm). From these data, it is clear that the desired ratiometric response is indeed being obtained, suggesting that **1**.TbEu has real potential as a luminescent probe to quantify oxygen concentrations in solution.

## Conclusion

The crux of the matter is that the delicate balance of energy transfer rates for—and possible conformations of—**1**.TbEu makes it a highly efficient, ratiometric, luminescence intensity based molecular reporter for dissolved oxygen concentration. The energy transfer processes involved in these systems are complex and subtle, but both isomeric systems studied here operate without the need to achieve pre-equilibrium. We would suggest that pre-equilibrium may require very short chromophore lanthanide separations if it is to be achieved. That said, we would caution that, in a bimetallic system, such close proximity of the chromophore to just one of the lanthanides would mean that the second lanthanide would be unlikely to be sensitized to any significant degree, meaning that both lanthanides would need to be equally close to the chromophore. Our approach described in this manuscript shows how sensitive bimetallic systems can be to small changes in distance relative to the sensitizing chromophore.

Our results also show that bimetallic complexes can be effective ratiometric probes for oxygen concentrations, and that small changes in structure can give rise to remarkable differences in behaviour. We are currently working to optimize these systems, extending our approach to different chromophores and to the ratiometric sensing of different analytes.
