# Supplementary material for: Bimetallic lanthanide complexes that display a ratiometric response to oxygen concentrations
Source: Chem Sci. 2015 Jan 12;6(3):2054–9. doi: 10.1039/c4sc03827d (PMC5654241; doi:10.1039/c4sc03827d)
Supplement: Supplementary file 1 [file SC-006-C4SC03827D-s001.pdf]

Electronic Supplementary Material (ESI) for Chemical Science.
This journal is © The Royal Society of Chemistry 2015

Supporting information for:

Bimetallic lanthanide complexes that display a ratiometric response to oxygen concentrations

Thomas Just Sørensen,[a,c] Alan M. Kenwright[c] and Stephen Faulkner[a]

Table of Content

Table of Content 1

Synthesis 2

Spectroscopy 8

Oxygen titrations 8

Estimating the dissolved oxygen concentrations .... **Error! Bookmark not defined.**

Conformational space available to the complexes 8

NMR spectra of lanthanide complexes 1-3 9

Photophysical properties of lanthanide complexes Ln.2 and Ln.3 11

Photophysical properties of lanthanide complexes 1.LnLn' 12

Luminescence lifetimes of the lanthanide complexes 1-3 14

Effect of oxygen concentration on the photophysics of lanthanide complexes 1.LnLn' 15

Time-resolved emission curves for 1.LnLn' 19

References 20

Synthesis

All materials were used as received; lanthanide trifluoromethanesulfonate salts were obtained from STREM and Sigma Aldrich in 98 % purity. NMR was performed on a Varian 700 MHz NMR spectrometer. Dialysis was performed using membranes from Harvard Apparatus UK.

Ln.**2** and Ln.**3** was prepared as previously reported.[1] The composition of Ln.2 and Ln.3 was verified by NMR and HRMS, see figures S1 and S2.

1.LnLn' was prepared using the Ugi reaction following the procedure we have used previously,[1b, 2] the inherent difficulty getting mass spectra of the multimetallic species did only allow us to characterize the molecule using NMR and luminescence, the data are given in figures S1-S11 below.

1.EuTb: Eu.**2** (536 mg, 0.9 mmol), Tb.**3** (537 mg, 0.87 mmol), naphthalene-2-carboxaldehyde (156.2 mg, 1 ,mol), benzyl isonitrile (117 mg, 1 mmol, ~150 μl), and sodium sulfate (1 g) was dissolved in ethanol (15 ml) and left to stir at 40 °C for 3 days. After this the solids were filtered off and the solvent removed in vacuo. The crude was taken in a minimum of methanol and precipitated with ether. The supernatant was decanted off, more ether was added, the solution was agitated, allowed to settle, and the supernatant was decanted off. The solvent was removed in vacuo. The crude was taken up in water (50 ml) and dialysed over a 500MwCO cellulose membrane (Spectrumlabs) for three days, with 6 water changes. After removing the solvent a waxy hydroscopic material was obtained. Repeated precipitation with ether from methanol allowed for the isolation of the product as a white powder in a yield of 120 mg (9.4 %), the material is highly hygroscopic, which prohibited quantitate analysis such as elemental analysis and ICP-MS. ^{1}H (D_2O) δ 256.08, 239.95, 202.18, 192.25, 187.41, 135.56 (m), 110.68, 102.65, 82.43 (m), 71.49, 57.32 (m), 49.70, 48.32, 33.67, 33.24, 32.00 (m), 31.69, 30.55, 18.29, 13.26 (m), 12.20 (m), 11.48 (m), 10.82 (m),9.81 (m), 8.31 (m), 8.23 (m), 8.13 (m), 7.98 (m), 7.94 (m), 7.83 (m),7.74 (m), 7.61 (m), 7.51 (m), 7.29 (m), 7.22 (m), 6.98 (m), 6.89 (m), 6.07 (m), 5.28 (m), 4.64, 4.33 (m), 4.26 (m), 4.18 (m), 3.97, 3.82, 3.59, 3.39, 3.23, 3.07, 3.12, 3.07, 2.99, 2.86, 2.69, 2.51, 2.27, 2.11, 1.78, 1.55 (m), 1.35 (m), 1.20, 0.75 (m), 0.52 (m), 0.04, -0.56, -1.17, -2.31, -2.82, -3.24, -4.34 (m), -5.13, -5.71, -6.77, -7.25, -7.83, -8.11 -9.50 (m), -10.92, -11.79, -12.68, -14.10, -14.32, -15.55, -16.16, -16.56, -37.60 (m), -49.23 (m), -62.38 (m), -67.43, -77.32, -78.49, -80.16, -95.30, -98.77, -101.01, -110.10, -114.36 , -122.65 , -199.10, -364.78, -376.03, -379.12, -393.09, -369.16 (m).

1.TbEu: Tb.**2** (536 mg, 0.89 mmol), Eu.**3** (531 mg, 0.87 mmol), naphthalene-2-carboxaldehyde (156.2 mg, 1 ,mol), benzyl isonitrile (117 mg, 1 mmol, ~150 μl), and sodium sulfate (1 g) was dissolved in ethanol (15 ml) and left to stir at 40 °C for 3 days. After this the solids were filtered off and the solvent removed in vacuo. The crude was taken in a minimum of methanol and precipitated with ether. The supernatant was decanted off, more ether was added, the solution was agitated, allowed to settle, and the supernatant was decanted off. The solvent was removed in vacuo. The crude was taken up in water (50 ml) and dialysed over a 500MwCO cellulose membrane (Spectrumlabs) for three days, with 6 water changes. After removing the solvent a waxy hydroscopic material was obtained. Repeated precipitation with ether from methanol

allowed for the isolation of the product as a white powder in a yield of 180 mg (14.1 %), the material is highly hygroscopic, which prohibited quantitate analysis such as elemental analysis and ICP-MS.^{1}H (D_2O) δ 258.48, 249.41, 245.76, 242.37, 201.32, 191.92, 135.68, 117.02, 108.88, 82.36, 76.28 (m), 73.56, 60.56 (m), 52.75, 45.32, 39.75, 33.70 (m), 33.05, 32.16, 30.92, 30.74, 23.87, 19.35, 13.29 (m), 12.32 (m), 11.76 (m), 11.01 (m), 8.25 (m), 7.34 (m), 7.28 (m), 5.52, 5.01, 4.65, 3.97, 3.82, 3.62, 3.41, 3.26, 3.13, 3.08, 2.93, 2.86, 2.72, 2.31, 2.15, 1.58 (m), 1.40 (m), 1.23 (m), 1.17 (m), 1.08 (m), 0.79 (m), 0.55 (m), 0.07 (m), -0.16, -0.48, -1.15, -1.51 (m), -1.91, -3.07, -3.35, -4.35 (m), -4.63 (m), -4.92, -5.65, -6.19 (m), -6.74, -6.99, -7.38 (m), -7.90, -8.10 (m), -8.84 (m), -9.47(m), -10.35, -11.16, -11.45 (m), -12.11, 12.73, -14.67, -14.73, -14.92, -15.03, -15.39, -16.16 (m), -16.61, -33.98 (m), -45.22 (m), -56.19 (m), -63.98, -69.77 (m), -70.92 (m), -75.54, -76.55, -78.24 (m), -81.28, -95.28, -96.56, -98.86, -103.93 (m), -108.69, -116.49, 130.07, -201.11 (m), -213.55 (m), -224.68 (m), -227.58 (m), -362.86, -373.57, -376.52, -396.24, -401.43.

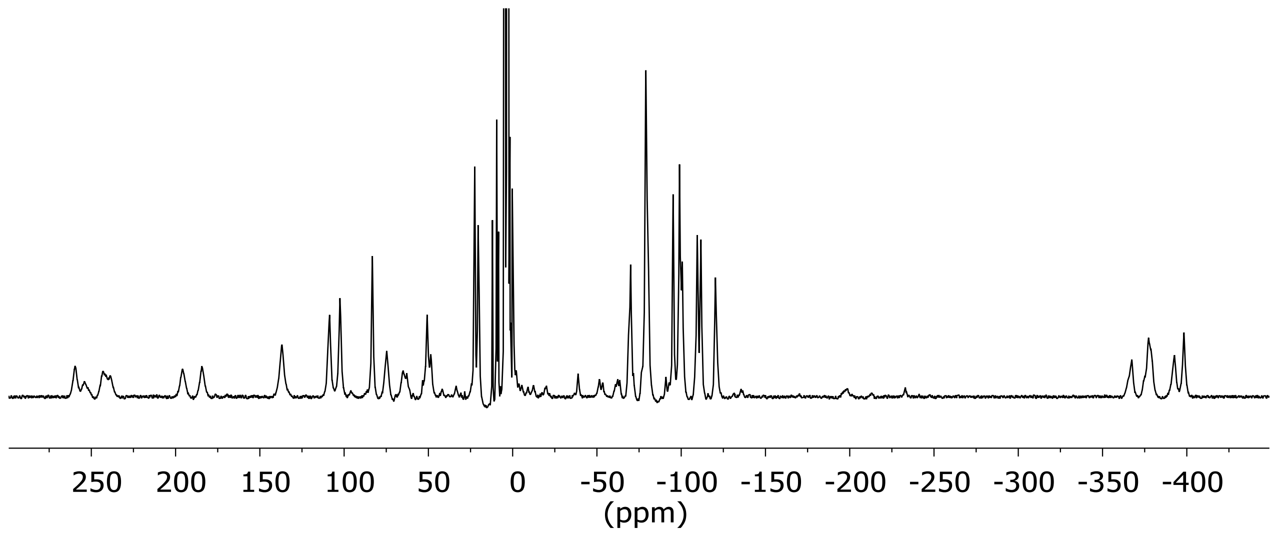

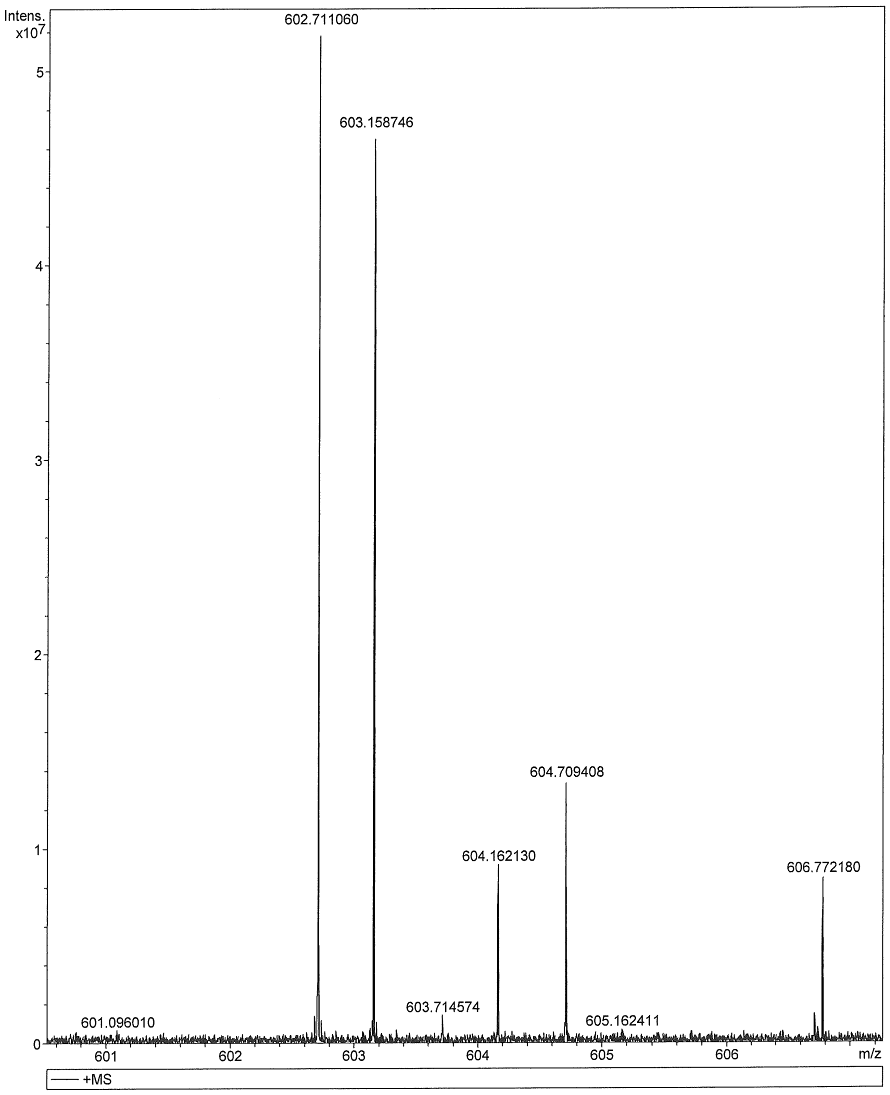

Figure S1a. Paramagnetic 700 MHz ^{1}H NMR spectra in D_2O employing water suppression through saturation (top) and HRMS spectra (bottom) of Tb.**2**.

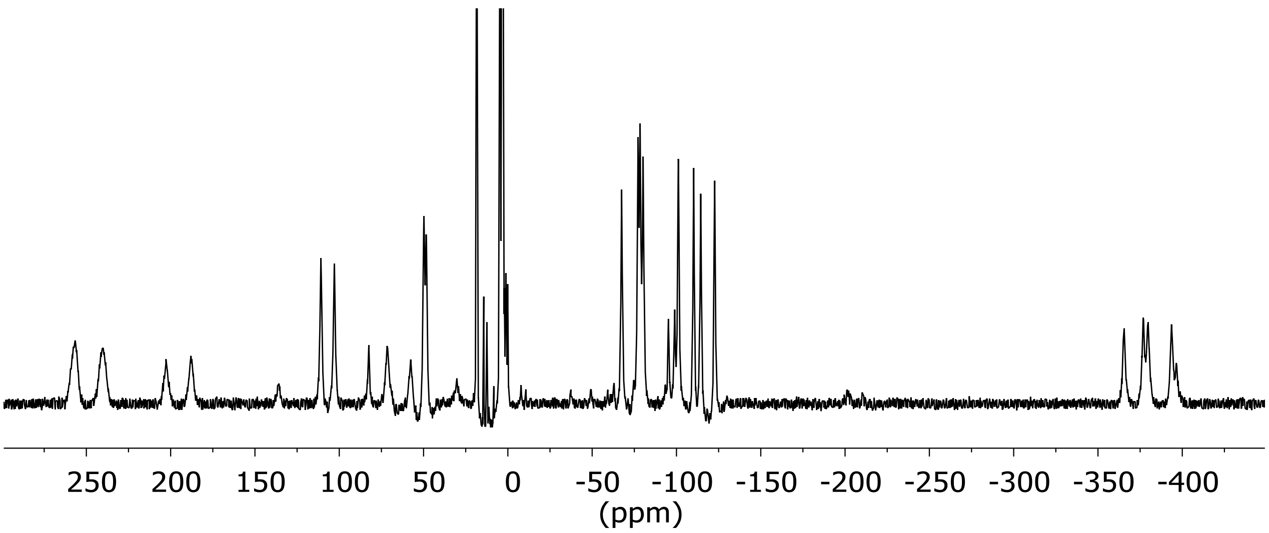

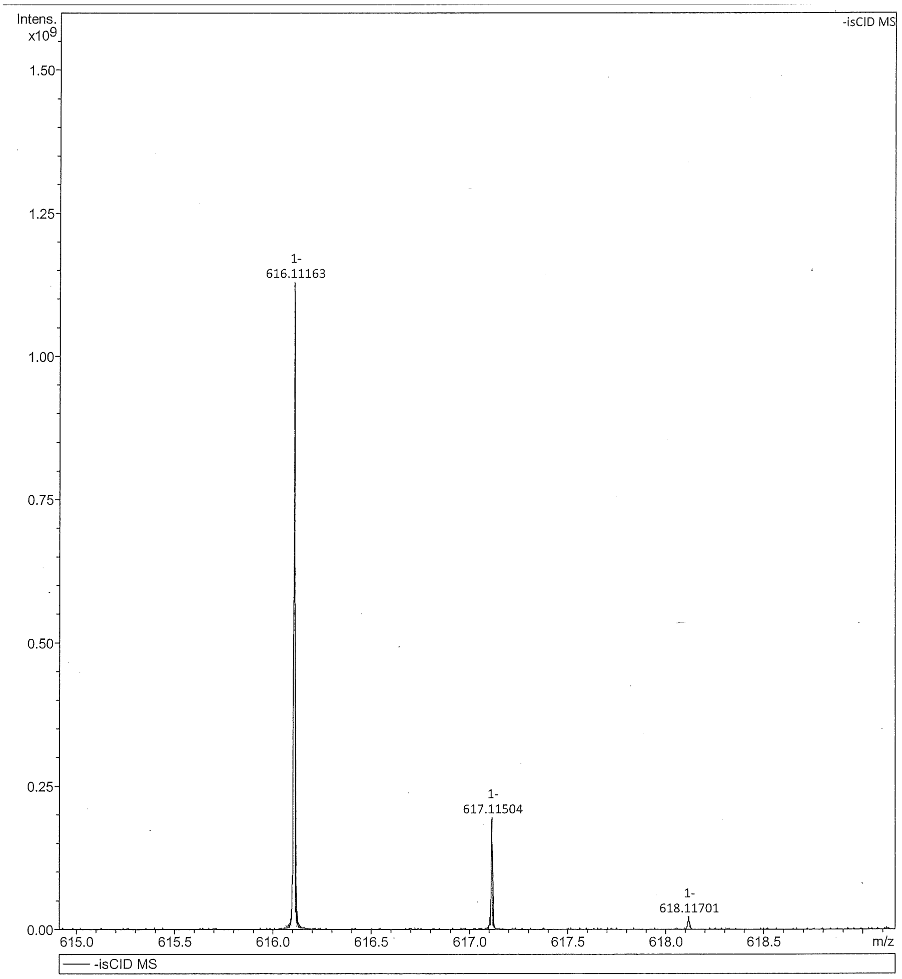

Figure S1b. Paramagnetic 700 MHz ^{1}H NMR spectra in D_2O employing water suppression through saturation (top) and HRMS spectra (bottom) of Tb.**3**.

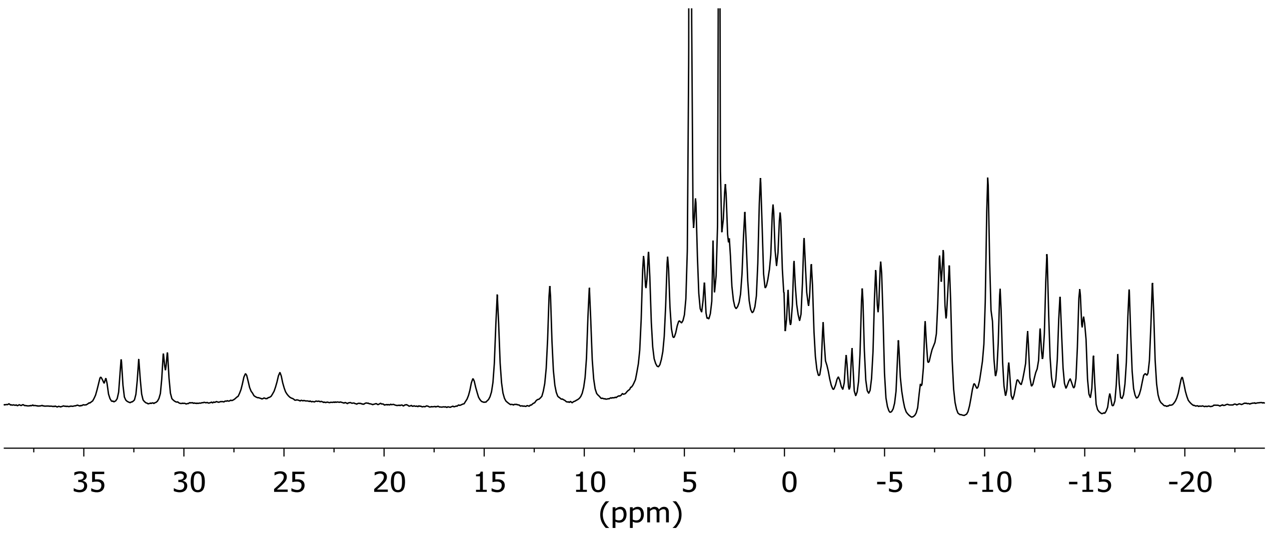

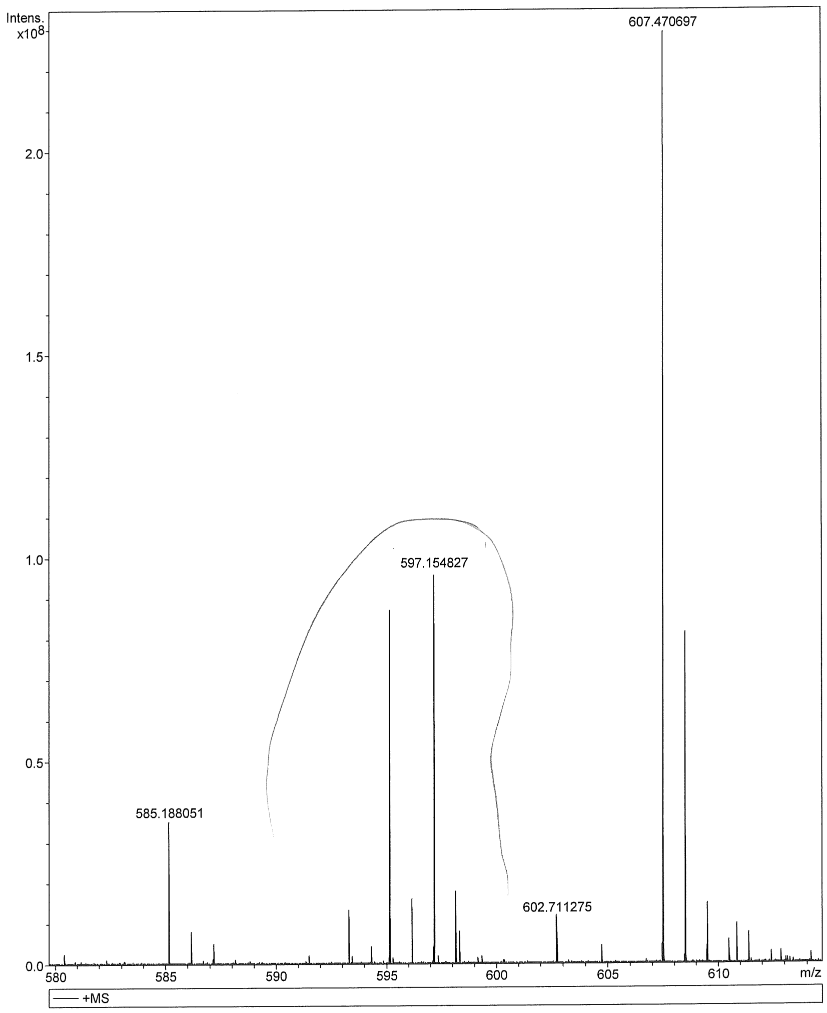

Figure S2a. Paramagnetic 400 MHz ^{1}H NMR spectra in D_2O employing water suppression through saturation (top) and HRMS spectra (bottom) of Eu.**2**.

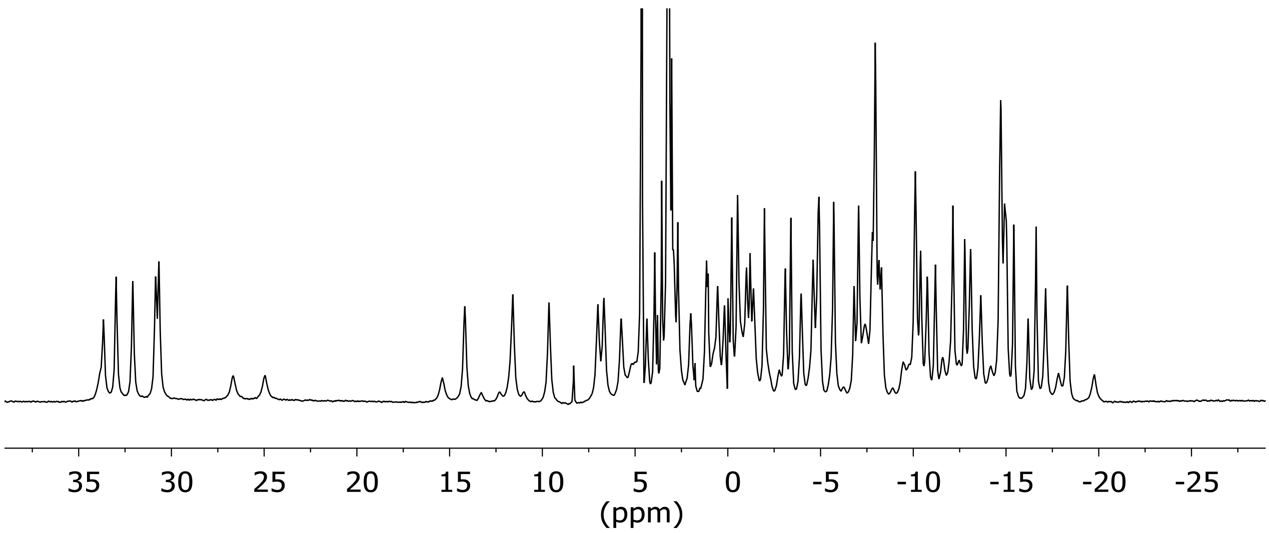

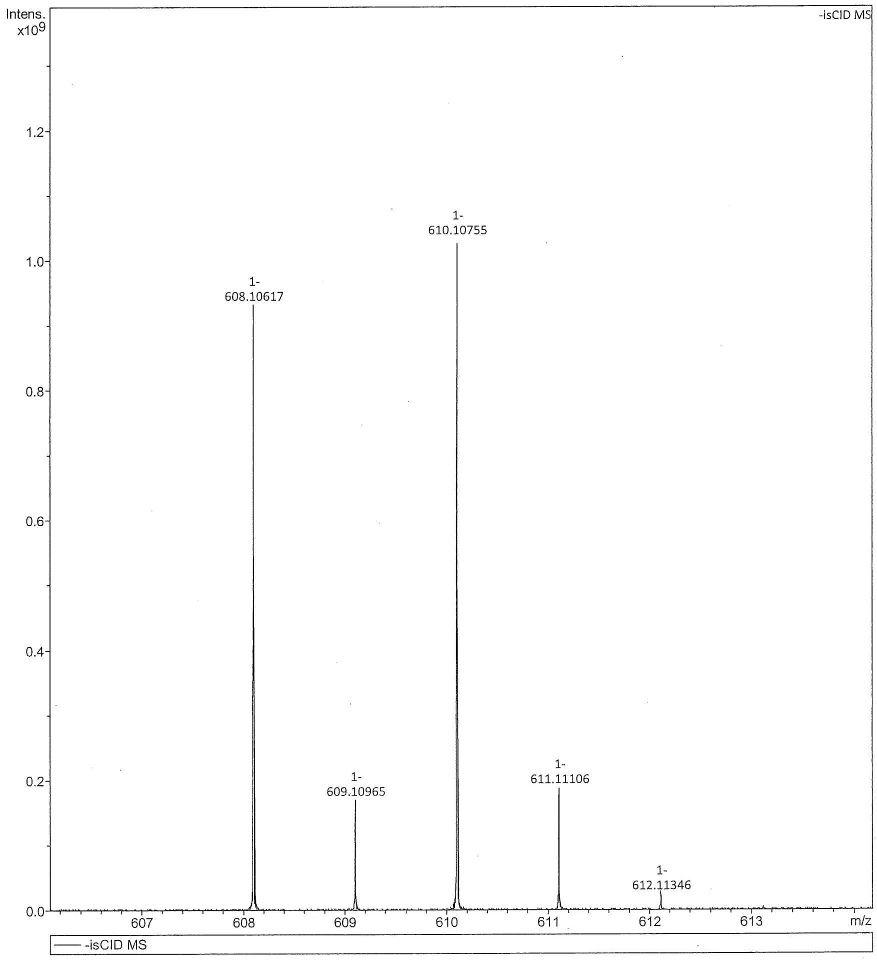

Figure S2b. Paramagnetic 700 MHz ^{1}H NMR spectra in D_2O employing water suppression through saturation (top) and HRMS spectra (bottom) of Eu.**3**.

Spectroscopy

Luminescence spectroscopy was performed using a Horiba Fluorolog 3.

Oxygen titrations

The effect of oxygen concentration was determined by measuring the photophysical properties at ambient conditions. At oxygen free conditions, by degassing the solution through 5 freeze pump thaw cycles. And at oxygen saturated conditions by backfilling with oxygen after a series of freeze pump thaw cycles.

Furthermore, an oxygen titration was carried out in pure water by degassing with N_2 or purging with O_2 and determining the dissolved oxygen concentration using an optical DO meter from Mettler-Toledo.

Conformational space available to the complexes

The terbium luminescence lifetime in H_2O/D_2O depends on the wavelength of excitation. Direct excitation yields a longer observed luminescence lifetime than sensitiser-mediated excitation. The tentative conclusion is that the conformational space available in the two excited state populations differ:

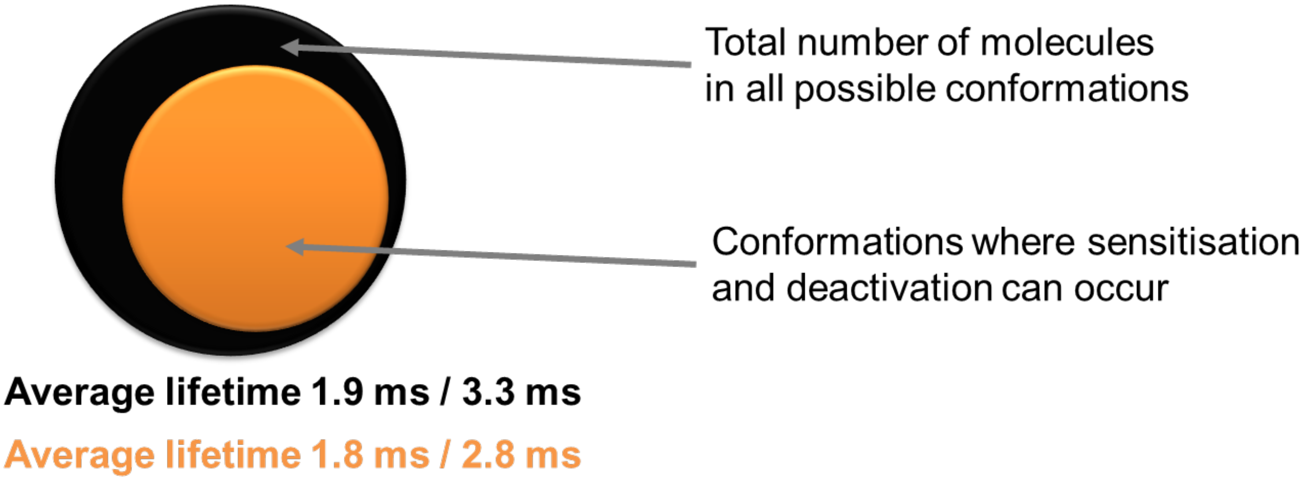

NMR spectra of lanthanide complexes 1-3

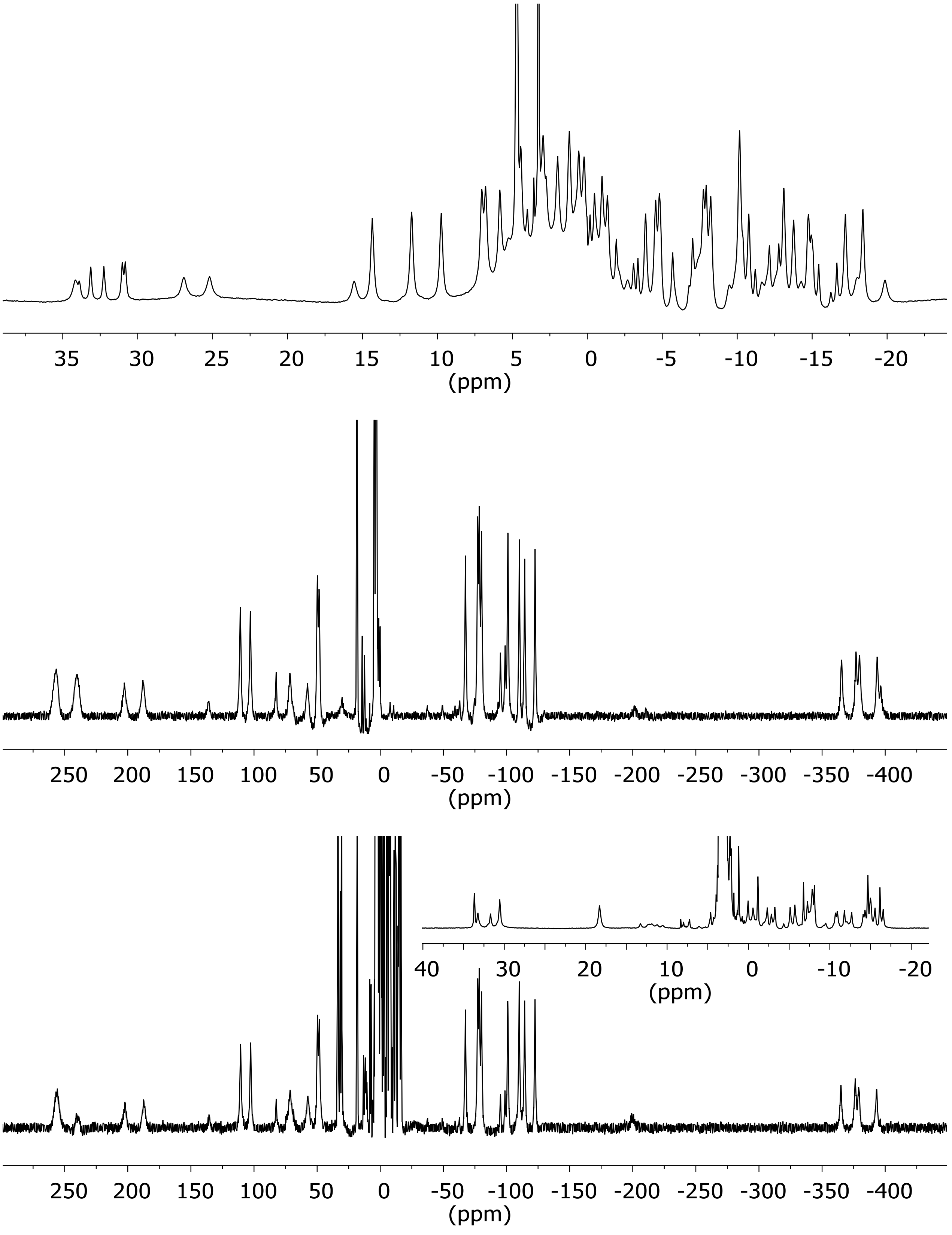

Figure S3. Paramagnetic ^{1}H NMR spectra in D_2O employing water suppression through saturation, from the top: Eu.**2** (400 MHz), Tb.**3** (700 MHz), and **1**.EuTb (700 MHz).

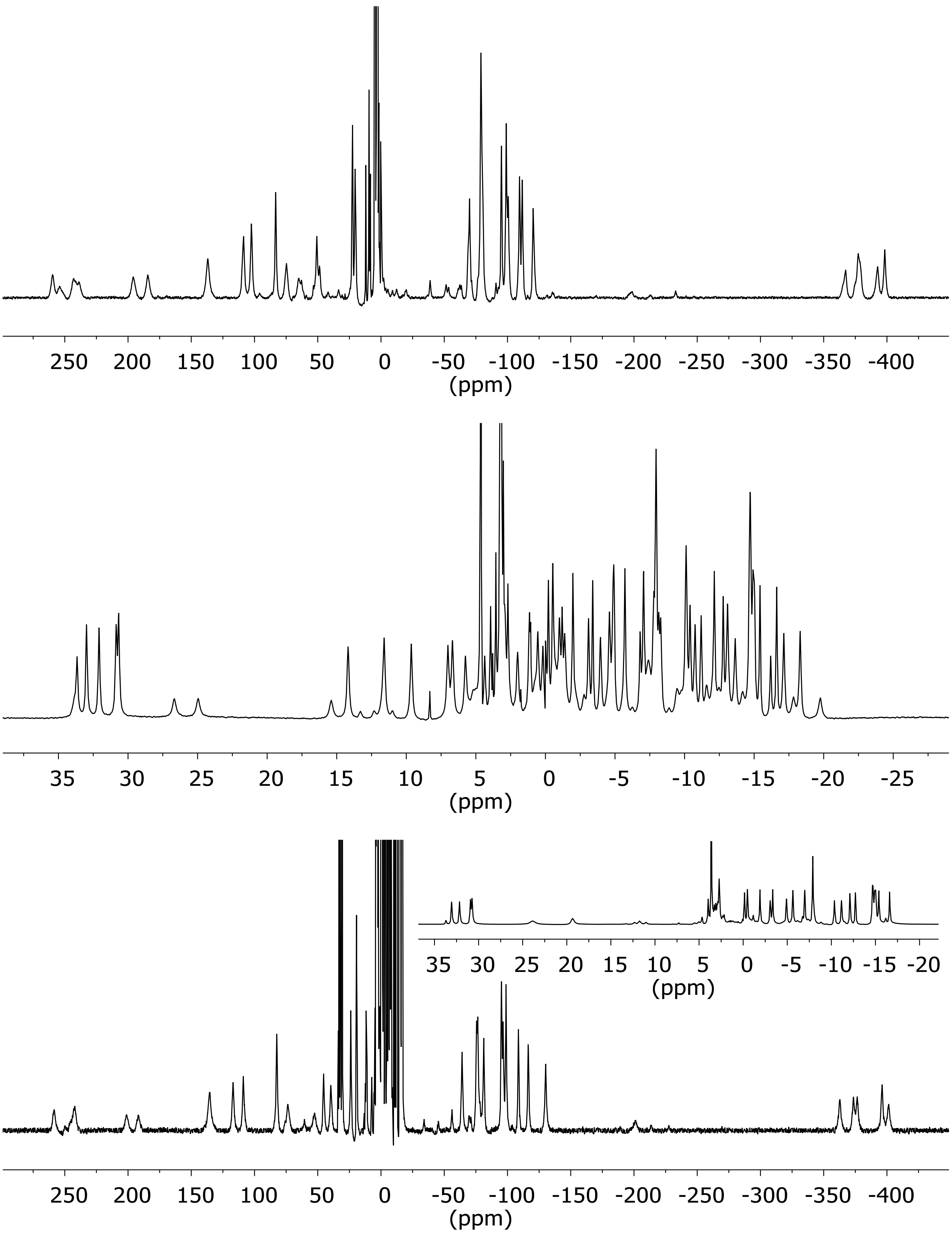

Figure S4. Paramagnetic ^{1}H NMR spectra in D_2O employing water suppression through saturation, from the top: Tb.**2** (700 MHz), Eu.**3** (700 MHz), and **1**.TbEu (700 MHz).

Photophysical properties of lanthanide complexes Ln.2 and Ln.3

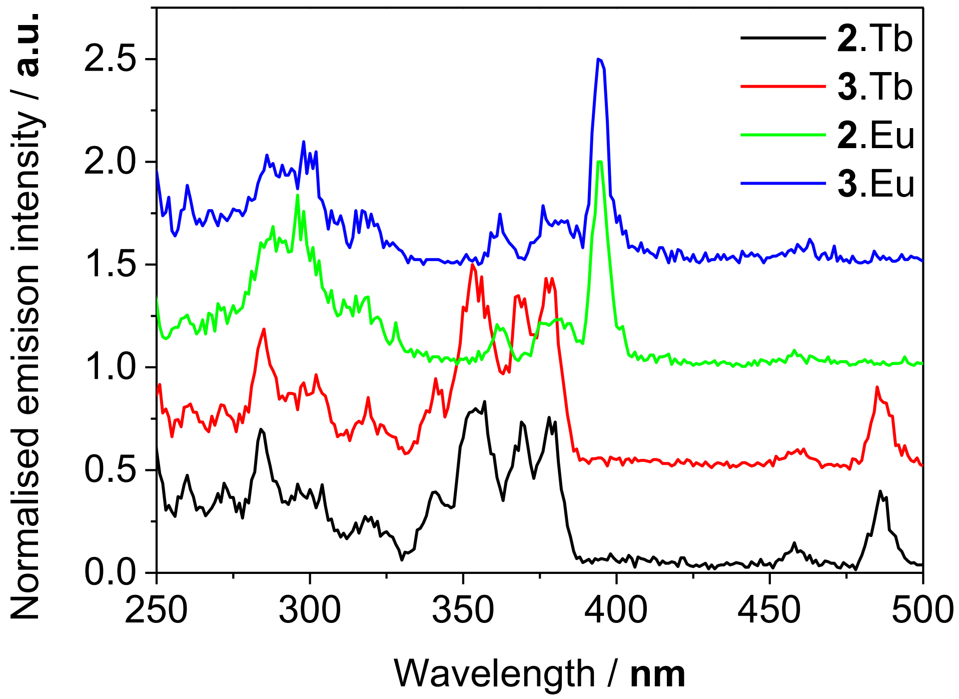

Figure S5. Corrected and normalized excitation spectra of Ln.**2** and Ln.**3** (Ln: Eu, Tb).

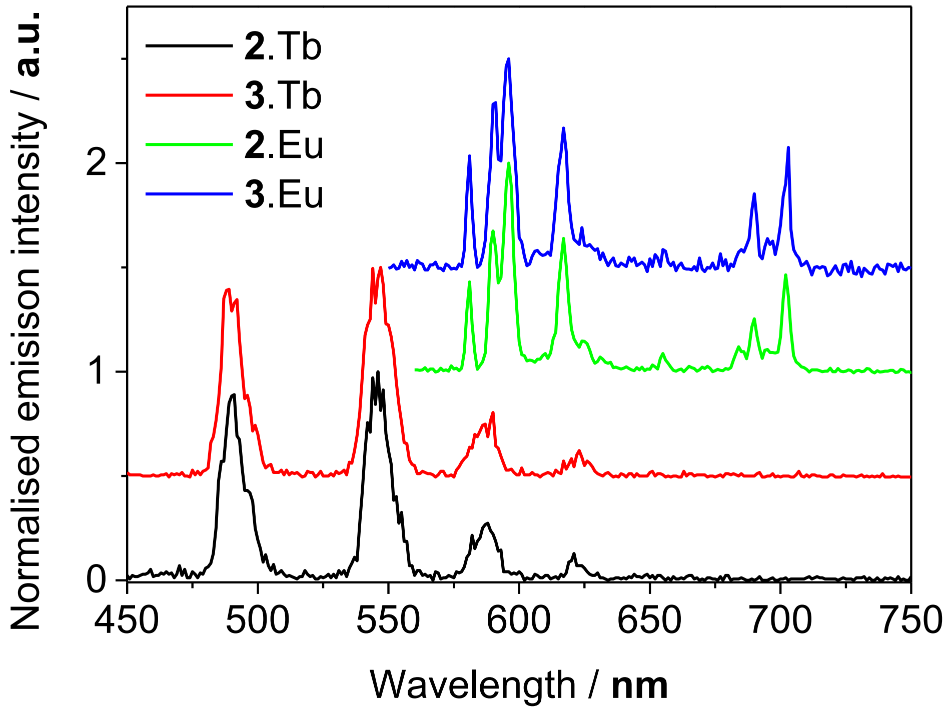

Figure S6. Normalized emission spectra of Ln.**2** and Ln.**3** (Ln: Eu, Tb).

Photophysical properties of lanthanide complexes 1.LnLn’

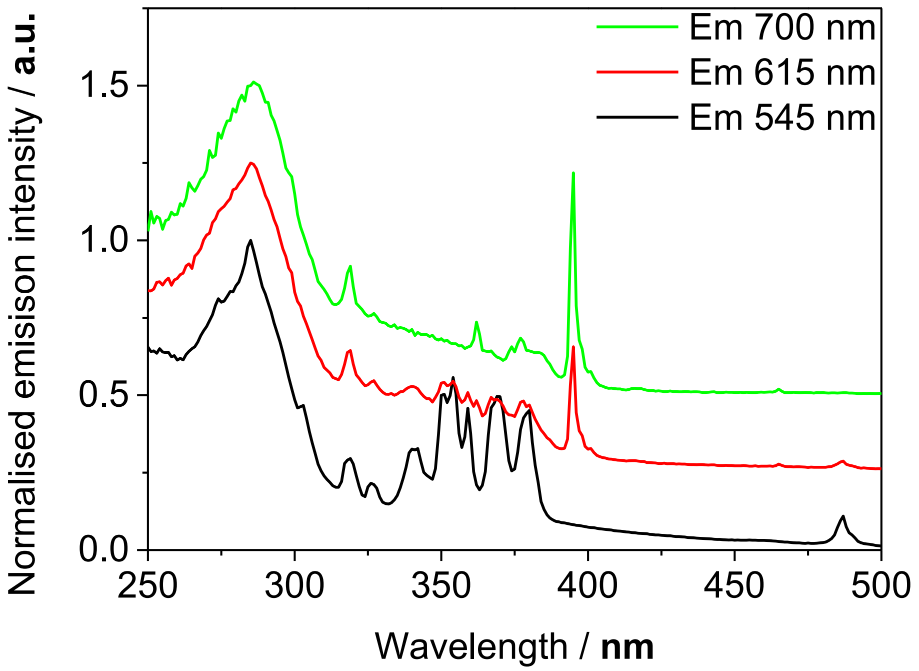

Figure S7. Corrected excitation spectra of **1**.EuTb monitored at different wavelengths; the spectra have been normalized at 285 nm.

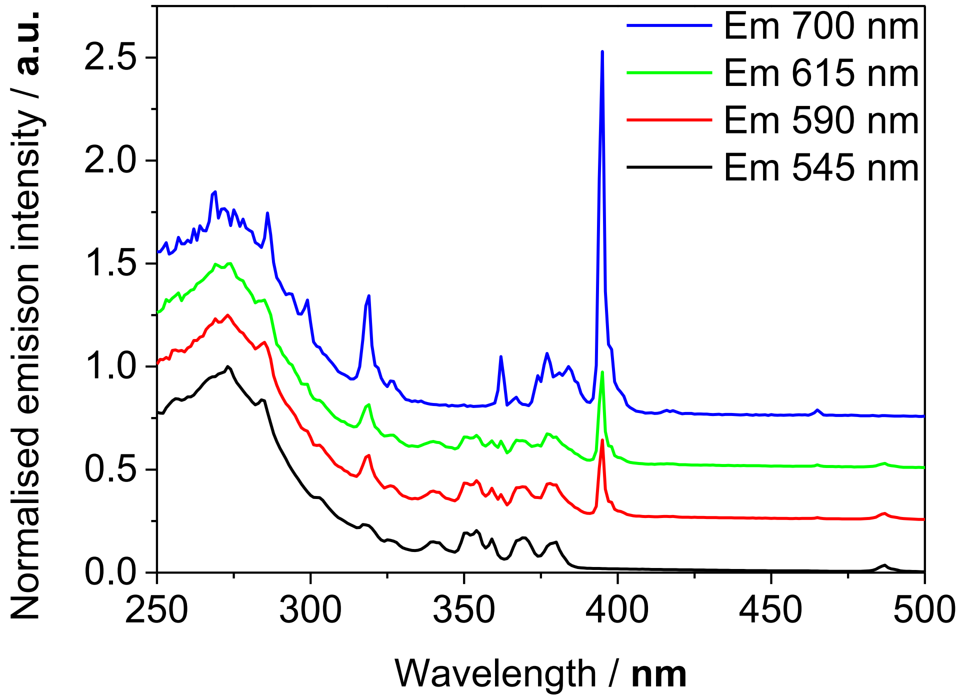

Figure S8. Corrected excitation spectra of **1**.TbEu monitored at different wavelengths; the spectra have been normalized at 273 nm.

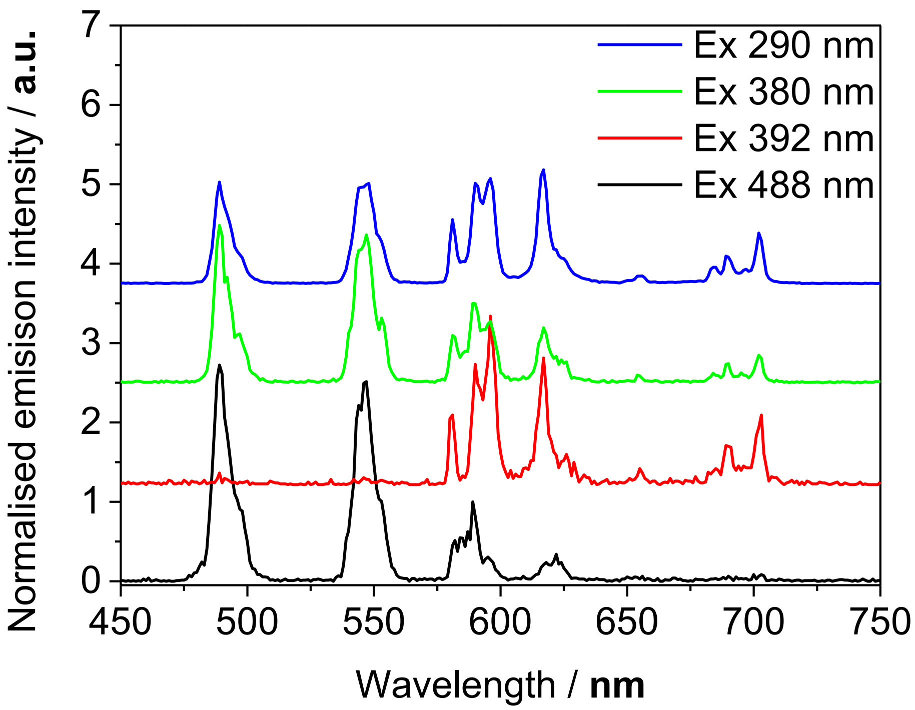

Figure S9. Uncorrected emission spectra of **1**.EuTb following excitation at different wavelengths; the spectra have been normalized at 589 nm.

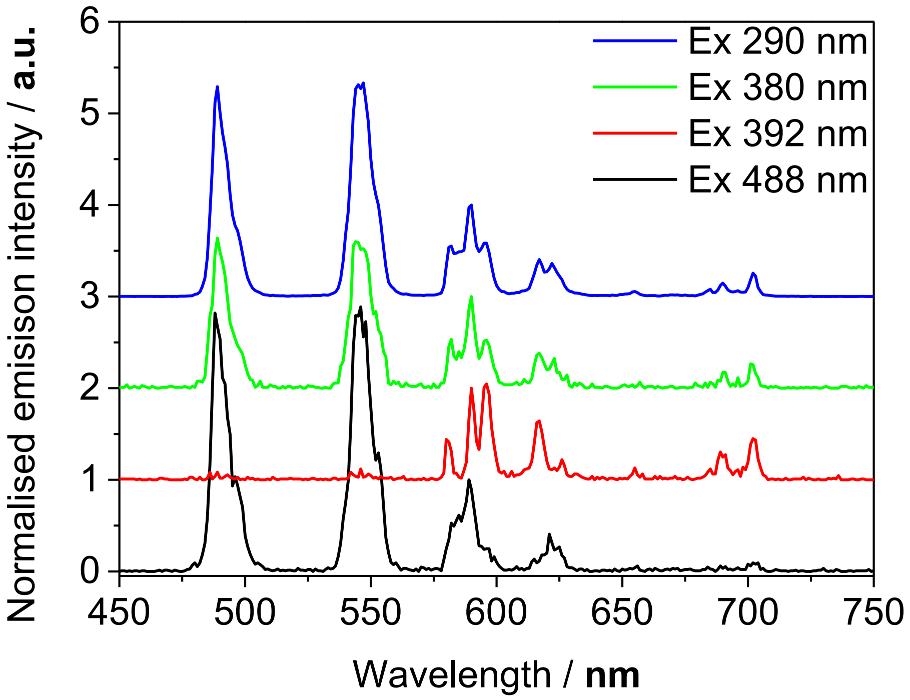

Figure S10. Uncorrected emission spectra of **1**.TbEu following excitation at different wavelengths; the spectra have been normalized at 589 nm.

Luminescence lifetimes of the lanthanide complexes 1-3

Table S1. Lifetimes in milliseconds of terbium and europium centered emission in **1**.EuTb at ambient, degassed and saturated O_2 concentration (ignoring the possible differences due to excitation in ligand vs. metal for Tb, N is the number of repetitions of the measurement, σ standard deviation).

solvent	O2	Tb	N	σ	Eu	N	σ
H_2O	Ambient	1.88	6	0.06	0.60	8	0.03
H_2O	Degassed	1.88	8	0.07	0.62	8	0.02
H_2O	Saturated	1.86	9	0.07	0.61	8	0.01
H_2O	all	1.87	23	0.06	0.61	24	0.02
D_2O	Ambient	3.15	6	0.26	2.10	8	0.03
q		0.8	-	-	1.1	-	-

Table S2. Lifetimes in milliseconds of terbium and europium centered emission in **1**.TbEu at ambient, degassed and saturated O_2 concentration (ignoring the possible differences due to excitation in ligand vs. metal for Tb, N is the number of repetitions of the measurement, σ standard deviation).

solvent	O2	Tb	N	σ	Eu	N	σ
H_2O	Ambient	1.78	9	0.08	0.64	11	0.01
H_2O	Degassed	1.80	9	0.09	0.63	6	0.01
H_2O	Saturated	1.79	8	0.13	0.63	6	0.005
H_2O	all	1.79	26	0.08	0.63	23	0.01
D_2O	Ambient	3.10	7	0.21	2.43	6	0.04
q		1.1	-	-	1.1	-	-

Table S3. Lifetimes in milliseconds of terbium and europium centered emission in Ln.**2** and Ln.**3** at ambient O_2 concentration (N is the number of repetitions of the measurement, σ standard deviation).

Complex	solvent	Tb	N	σ	Eu	N	σ
Ln.**2**	H_2O	1.45	9	0.5	0.64	5	0.04
	D_2O	3.06	9	0.4	2.32	6	0.09
q		1.5	-	-	1.3	-	-
Ln.**3**	H_2O	1.74	8	0.2	0.68	6	0.06
	D_2O	3.07	8	0.3	2.34	9	0.2
q		0.9	-	-	1.2	-	-

Effect of oxygen concentration on the photophysics of lanthanide complexes 1.LnLn'

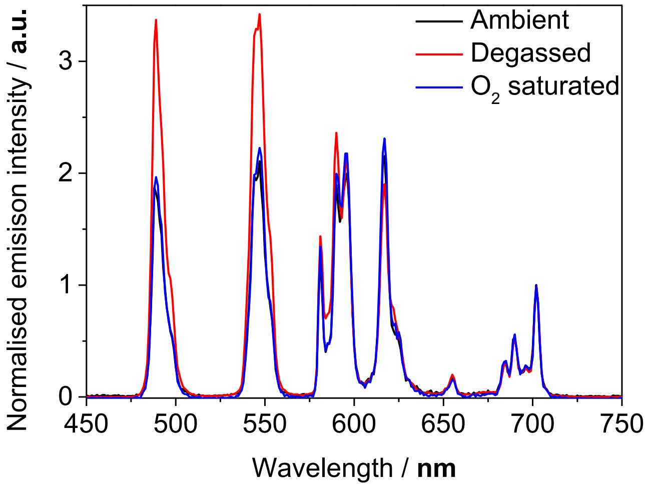

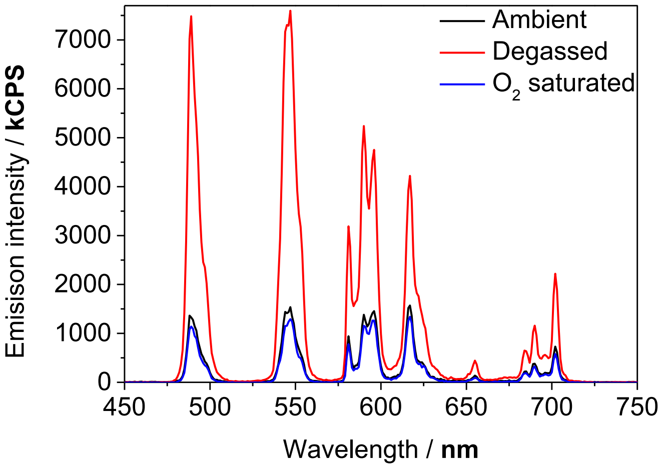

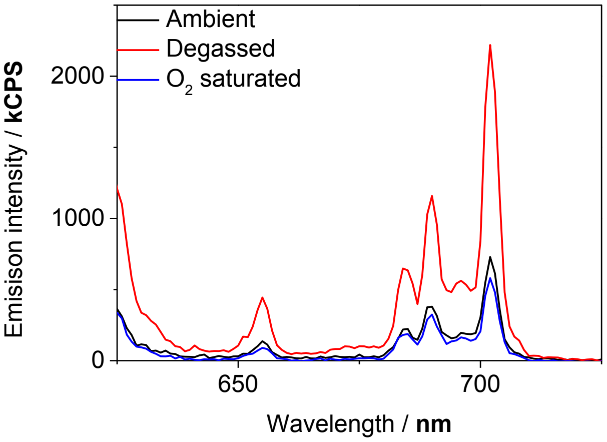

Figure S11. Uncorrected emission spectra of **1**.EuTb following excitation at 290 nm; the spectra have been normalized at 702 nm.

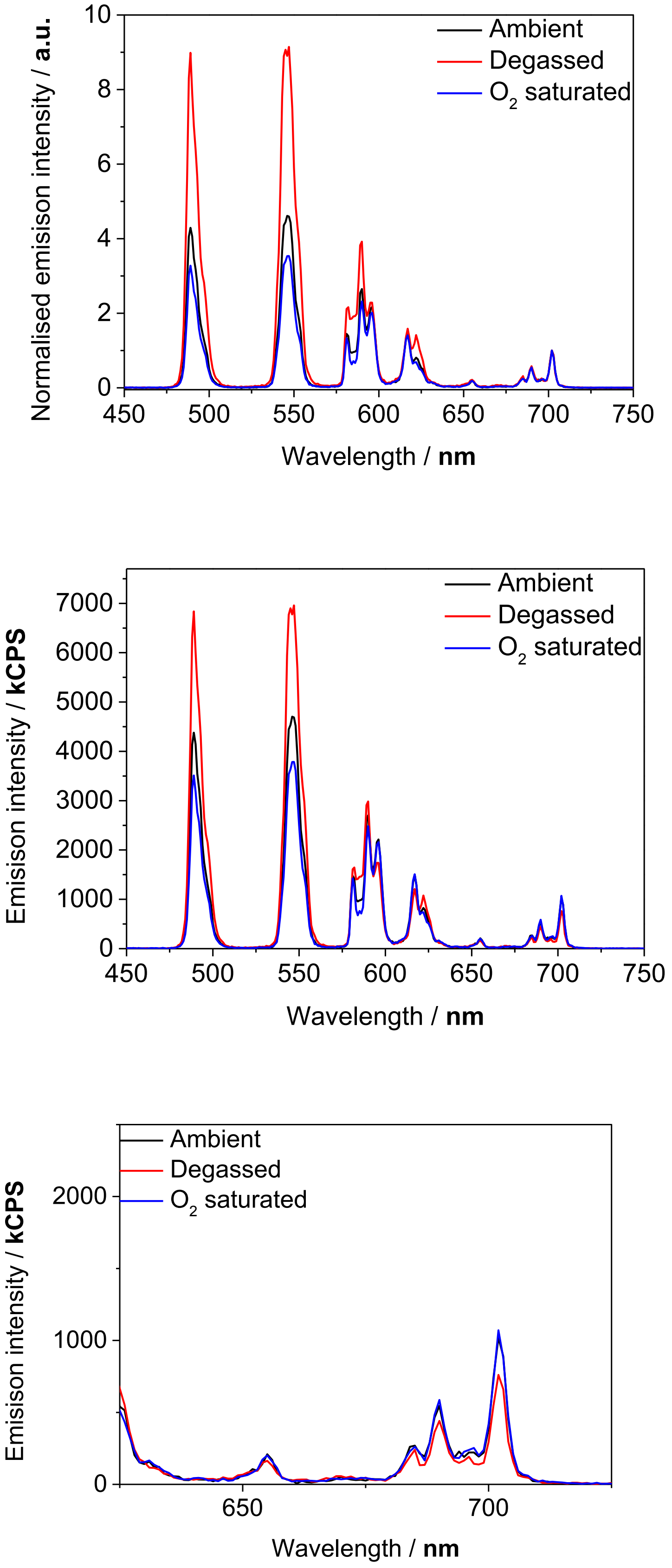

Figure S12. Uncorrected emission spectra of **1**.TbEu following excitation at 290 nm; the spectra have been normalized at 702 nm.

Table S4 Full table of lifetimes of terbium and europium centred emission in **1**.EuTb at ambient, degassed and saturated o2 concentration.

Ex\Em			488 nm (Tb)	545 nm (Tb)	590 nm	615 nm	700 nm (Eu)
	solvent	O2					
290 nm	H_2O	Ambient	1.80	1.79	1.7 (1.2)<br>0.63 (6.0)	1.1 (2.0)<br>0.49 (3.2)	0.62
	H_2O	Degassed	0.27 (3.8)<br>1.93 (3.8)	0.25 (3.0)<br>1.81 (3.4)	1.59 (1.5)<br>0.49 (3.9)	0.66	0.62
	H_2O	Saturated	1.77	1.78	1.4 (2.3)<br>056 (5.8)	0.83 (4.0)<br>0.33 (2.4)	0.63
	D_2O	Ambient	2.88	2.75	2.24	2.09	2.07
380 nm	H_2O	Ambient	1.91	1.91	1.75 (1.2)<br>0.53 (1.3)	1.44 (0.5)<br>0.50 (6.4)	0.59
	H_2O	Degassed	1.95	1.94	1.87 (1.87)<br>0.58 (2.4)	1.43 (0.9)<br>0.43 (1.3)	0.60
	H_2O	Saturated	1.89	1.93	1.89 (1.8)<br>0.62 (2.3)	1.35 (0.8)<br>0.43 (1.5)	0.60
	D_2O	Ambient	3.30	3.37	2.44	2.11	2.12
392 nm (Eu)	H_2O	Ambient	-	1.64	0.63	0.61	0.58
	H_2O	Degassed	-	1.88	0.62	0.61	0.63
	H_2O	Saturated	1.90	1.78	062	0.61	0.62
	D_2O	Ambient	-	3.0	2.16	2.11	2.10
488 nm (Tb)	H_2O	Ambient	1.91	1.95	1.47	1.30	0.55
	H_2O	Degassed	1.82	1.81	1.19	1.03	06.1
	H_2O	Saturated	1.92	1.94	1.51	1.30	0.60
	D_2O	Ambient	3.29	3.29	2.83	2.53	2.07

Table S5. Full table of lifetimes of terbium and europium centred emission in **1**.TbEu at ambient, degassed and saturated o2 concentration.

Ex\Em			488 nm (Tb)	545 nm (Tb)	590 nm	615 nm	700 nm (Eu)
	solvent	O2					
290 nm	H_2O	Ambient	1.71	1.70	1.6 (3.5)<br>0.58 (5.8)	1.6 (0.9)<br>0.61 (3.3)	0.63
	H_2O	Degassed	0.18 (2.1)<br>1.8 (7.7)	0.15 (1.6)<br>1.7 (4.7)	1.6 (5.7)<br>0.43 (4.6)	1.6 (1.3)<br>0.53 (2.8)	0.62
	H_2O	Saturated	1.7	1.67	1.5 (3.2)<br>0.57 (5.5)	1.2 (1.5)<br>0.51 (2.8)	0.63
	D_2O	Ambient	2.85	2.76	2.59	2.56	2.39
380 nm	H_2O	Ambient	1.83	1.85	-	1.9 (0.5)<br>0.63 (1.2)	0.62
	H_2O	Degassed	1.85	1.87	1.8 (1.6)<br>0.6 (2.0)	2.0 (0.4)<br>0.65 (1.2)	0.62
	H_2O	Saturated	1.84	1.83	-	1.6 (0.9)<br>0.54 (1.5)	0.63
	D_2O	Ambient	3.25	3.26	3.0 (2.8)<br>2.1 (1.2)	2.54	2.49
392 nm (Eu)	H_2O	Ambient	-	1.7	0.65	0.63	0.63
	H_2O	Degassed	-	1.6	0.64	0.63	0.62
	H_2O	Saturated	-	1.7	0.64	0.63	0.63
	D_2O	Ambient	-	3.06	2.45	2.39	2.41
488 nm (Tb)	H_2O	Ambient	1.79	1.86	1.96	1.9 (1.6)<br>0.6 (0.7)	0.65
	H_2O	Degassed	1.84	0.22 (0.5)<br>1.87 (3.7)	1.8 (3.7)<br>0.47 (0.6)	1.8 (1.4)<br>0.5 (0.6)	0.63
	H_2O	Saturated	1.82	1.85	1.7 (3.3)<br>0.4 (0.5)	1.9 (1.6)<br>0.61 (0.8)	0.64
	D_2O	Ambient	3.25	3.24	3.0	2.82	2.45

Time-resolved emission curves for 1.LnLn'

Figure S13. Lifetime traces for **1**.EuTb

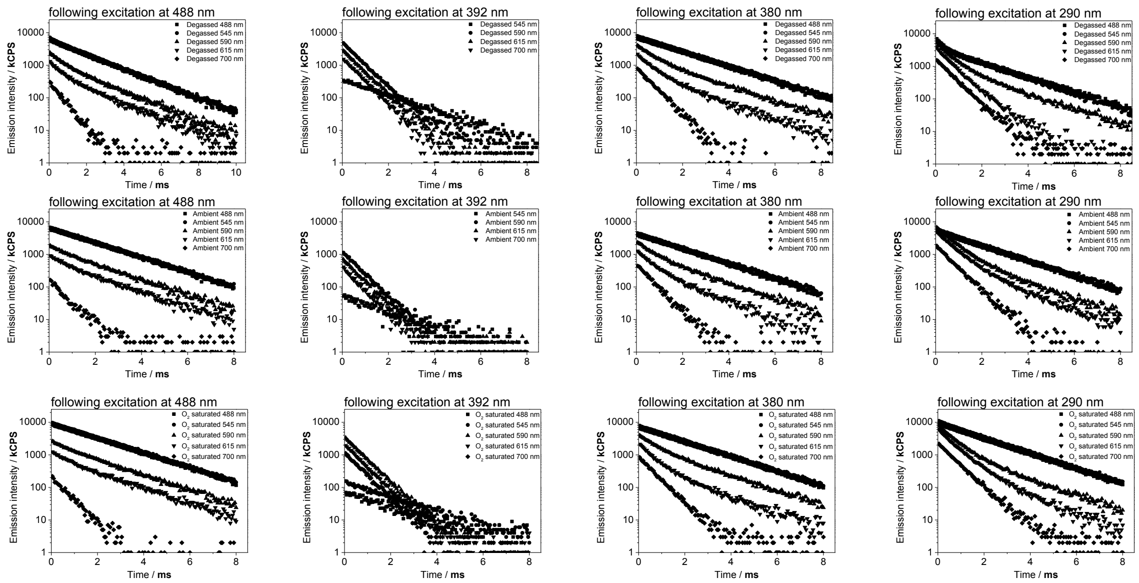

Figure S14 Lifetime traces for **1**.TbEu

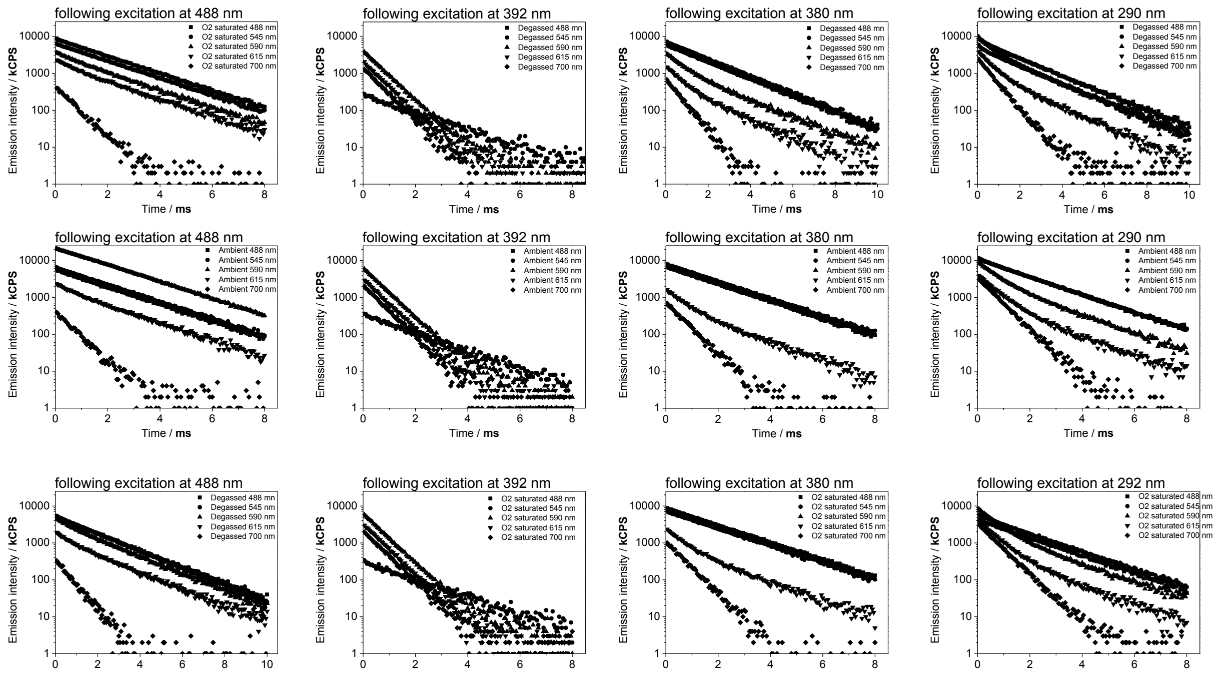

References

1. (a) Perry, W. S.; Pope, S. J. A.; Allain, C.; Coe, B. J.; Kenwright, A. M.; Faulkner, S., Synthesis and photophysical properties of kinetically stable complexes containing a lanthanide ion and a transition metal antenna group. *Dalton Transactions* **2010,** *39* (45), 10974-10983; (b) Sørensen, T. J.; Tropiano, M.; Blackburn, O. A.; Tilney, J. A.; Kenwright, A. M.; Faulkner, S., Preparation and study of an f,f,f[prime or minute],f[prime or minute][prime or minute] covalently linked tetranuclear hetero-trimetallic complex - a europium, terbium, dysprosium triad. *Chem. Comm.* **2013,** *49* (8), 783-785.

2. Main, M.; Snaith, J. S.; Meloni, M. M.; Jauregui, M.; Sykes, D.; Faulkner, S.; Kenwright, A. M., Using the Ugi multicomponent condensation reaction to prepare families of chromophore appended azamacrocycles and their complexes. *Chemical Communications* **2008,** (41), 5212-5214.
